# Involvement of microRNA-related regulatory pathways in the glucose-mediated control of *Arabidopsis* early seedling development

**DOI:** 10.1093/jxb/ert239

**Published:** 2013-08-30

**Authors:** Gustavo Turqueto Duarte, Cleverson Carlos Matiolli, Bikram Datt Pant, Armin Schlereth, Wolf-Rüdiger Scheible, Mark Stitt, Renato Vicentini, Michel Vincentz

**Affiliations:** ^1^Laboratório de Genética de Plantas, Centro de Biologia Molecular e Engenharia Genética, Universidade Estadual de Campinas, CEP 13083-875, CP 6010; Campinas, São Paulo, Brazil; ^2^Max-Planck Institute of Molecular Plant Physiology, Am Mühlenberg 1, 14476 Potsdam-Golm, Germany; ^3^Plant Biology Division, The Samuel Roberts Noble Foundation, Ardmore, OK 73401, USA; ^4^Laboratório de Bioinformática e Biologia de Sistemas, Centro de Biologia Molecular e Engenharia Genética, Universidade Estadual de Campinas, CEP 13083-875, CP 6010; Campinas, São Paulo, Brazil; ^5^Departamento de Biologia Vegetal, Instituto de Biologia, CEP 13083-875, CP 6009; Campinas, São Paulo, Brazil

**Keywords:** Abscisic acid, AGO1, DCL1, glucose, HYL1, miRNA, plant development, post-transcriptional control, seed germination.

## Abstract

In plants, sugars such as glucose act as signalling molecules that promote changes in gene expression programmes that impact on growth and development. Recent evidence has revealed the potential importance of controlling mRNA decay in some aspects of glucose-mediated regulatory responses suggesting a role of microRNAs (miRNAs) in these responses. In order to get a better understanding of glucose-mediated development modulation involving miRNA-related regulatory pathways, early seedling development of mutants impaired in miRNA biogenesis (*hyl1-2* and *dcl1-11*) and miRNA activity (*ago1-25*) was evaluated. All mutants exhibited a glucose hyposensitive phenotype from germination up to seedling establishment, indicating that miRNA regulatory pathways are involved in the glucose-mediated delay of early seedling development. The expression profile of 200 miRNA primary transcripts (pri-miRs) was evaluated by large-scale quantitative real-time PCR profiling, which revealed that 38 pri-miRs were regulated by glucose. For several of them, the corresponding mature miRNAs are known to participate directly or indirectly in plant development, and their accumulation was shown to be co-regulated with the pri-miR by glucose. Furthermore, the expression of several miRNA target genes was found to be deregulated in response to glucose in the miRNA machinery mutants *ago1-25*, *dcl1-11*, and *hyl1-2*. Also, in these mutants, glucose promoted misexpression of genes for the three abscisic acid signalling elements ABI3, ABI4, and ABI5. Thus, miRNA regulatory pathways play a role in the adjustments of growth and development triggered by glucose signalling.

## Introduction

Photosynthesis-derived sugars are the main source of carbon skeletons and energy in plants. It is not surprising that glucose (Moore *et al*., 2003; Li *et al*., 2006), fructose (Cho and Yoo, 2011; Li *et al*., 2011), sucrose (Vaughn *et al*., 2002), and trehalose-6-phosphate (Delatte *et al*., 2011; Wahl *et al*., 2013) are important signalling molecules that impact on gene expression and several aspects of plant growth and development (e.g. germination, flowering, senescence; Smeekens *et al*., 2010; Eveland and Jackson, 2012; Stitt and Zeeman, 2012; Wahl *et al*., 2013). Integration of metabolic and circadian signals with environmental cues seems to coordinate starch degradation and sugar pools, thus impacting on carbon availability for growth (Stewart *et al*., 2011; Stitt and Zeeman, 2012). Sugar signalling also depends on the integration with other nutrient levels, such as phosphate, sulphate, and nitrogen, which are tightly related with circadian regulation and are required for proper plant development expression programmes (Rolland *et al*., 2006). In addition, sugars such as sucrose or glucose have been shown to interact with the hormones abscisic acid (ABA), ethylene (Yanagisawa *et al*., 2003; Karve *et al*., 2012), auxin, and cytokinin (Moore *et al*., 2003; Sairanen *et al*., 2012) in regulating plant development (Rolland *et al*, 2006) and stress responses (Price *et al*., 2003; Jossier *et al*., 2009).

The only sugar sensor that has been described unambiguously up to now is hexokinase 1 (HXK1), which senses glucose (Moore *et al*., 2003; Karve *et al*., 2012) and directly mediates the glucose-dependent transcriptional repression of the chlorophyll a/b-binding protein 2 gene (*CAB2*; Cho *et al*., 2006). Further evidence for transcriptional control mediating glucose responses comes from the observation that the APETALA2-type transcription factor ABI4, a component of the ABA signalling pathway, is involved in sugar-induced gene expression changes (Li *et al*., 2006; Bossi *et al*., 2009). In addition, post-transcriptional regulation is also involved in sugar signalling. For instance, evidence was found for sucrose affecting the translation of the mRNA for five *Arabidopsis thaliana* S-group bZIP-type transcription factors (Rahmani *et al*., 2009), and the degradation of the ethylene insensitive 3 (EIN3) transcription factor has been found to be glucose induced (Yanagisawa *et al*., 2003). Moreover, it was shown that glucose modulates the mRNA stability of the *Arabidopsis* transcription factor *AtbZIP63* as part of a regulatory scheme integrating energetic and abiotic stress (Matiolli *et al*., 2011). Moreover, the expression of *AtbZIP63* was found to be deregulated in the miRNA-processing mutant *hyl1-2* (Matiolli *et al*., 2011). This latest observation begs the question of whether miRNA-related regulatory pathways participate in glucose responses. *MIRNA* genes are transcribed by RNA polymerase II, resulting in a primary miRNA transcript (pri-miR) that is first converted into a precursor miRNA (pre-miRNA) by the endonuclease DICER-like1 (DCL1). Subsequently, the C2H2-zinc finger protein SERRATE (SE), the double-stranded RNA-binding protein HYPONASTIC LEAVES1 (HYL1), and DCL1 interact to trim the pre-miRNA generating a 21-nucleotide double-stranded miRNA/miRNA* duplex that is exported to the cytoplasm through the action of the exportin HASTY and other factors (Voinnet, 2009). Methylation of the duplex fragment on the 3′-terminal nucleotides of each strand by HEN protects the fragment from degradation by exonucleases. The mature miRNA is recognized by ARGONAUTE1 (AGO1) which is in turn integrated into the RNA-induced silencing complex (RISC) through a process that might also require HYL1 activity (Eamens *et al*., 2009). miRNA complementarity to specific mRNA guides RISC-mediated target mRNA cleavage and/or translational repression (Mi *et al*., 2008; Voinnet, 2009).

Germination and seedling establishment are crucial initial steps for plant development. It is well established that high exogenous glucose delays these early plant developmental phases (Arenas-Huertero *et al*., 2000; Cheng *et al*., 2002; Gibson, 2005; Dekkers *et al*., 2008). In the current work, sensitivity to glucose during seedling development was examined to analyse the capacity of mutants *hyl1-2* and *dcl1-11*, which are defective in pre-miRNA processing, and mutant *ago1-25*, which is impaired in miRNA activity, to modulate glucose responses. Young seedlings of these mutants showed a glucose hyposensitive phenotype, which was defined by the reduced capacity of glucose to delay early seedling development. This phenotype was correlated with the regulation of the expression of a set of 38 pri-miRs by 4% glucose, indicating that miRNA-related regulatory processes modulate the glucose-dependent delay of seedling development.

## Materials and methods

### Plant material and growth conditions

*A. thaliana* ecotype Columbia-0 (Col-0) was used as a wild-type control for the mutants ABA insensitive *abi4-1* (Finkelstein, 1994), ABA-deficient *aba2-1* (Schwartz *et al*., 1997), *ago1-25* (Morel *et al*., 2002), *dcl1-11* (Zhang *et al*., 2008), and *hyl1-2* (Vazquez *et al*., 2004) and Landsberg *erecta* (L*er*) was used as a wild-type control for the glucose-insensitive HXK1 knockout *gin2-1* (Moore *et al*., 2003). Seeds were surface sterilized and sown in plates with half-strength Murashige and Skoog media (MS/2) containing 0.5% (w/v) agar (type E) with 1–6% glucose, 4% mannitol, or 0.5–5 µM ABA (all Sigma-Aldrich reagents). The plates were placed in the dark at 4 °C for 2 d for stratification and then incubated at 24 °C under continuous light (50 µmol m^–2^ s^–1^) for up to 14 d. For phenotypic assays, each plate contained at least 20 seeds of each specified genotype and the assays were carried out in duplicate or triplicate. Analysis of deviance (McCullagh and Nelder, 1989) was used for statistical evaluation of germination and development efficiencies. For gene expression analysis assays, 5mg of seeds of each evaluated genotype was plated in at least triplicate.

### RNA isolation and cDNA synthesis

Total RNA was isolated following a LiCl-based protocol for seeds (Oñate-Sánchez and Vicente-Carbajosa, 2008), except for the addition of 3 µl of glycogen (20mg ml^–1^, Roche) in the last precipitation step. For pri-miR profiling, total RNA was treated with TURBO DNA-free (Ambion), according to the manufacturer’s instruction. For other quantitative real-time PCR (qRT-PCR) analyses, DNAse treatment was only carried out in the cases where the primers pairs could not be designed to span exon–exon junctions. Synthesis of cDNA was carried out with ImProm II Reverse Transcriptase (Promega), according to manufacturer instructions.

### qRT-PCR analysis

Gene expression analysis by qRT-PCR was conducted as described by Czechowski *et al*. (2005) and Udvardi *et al*. (2008), evaluating gene expression with the Delta-Delta Ct method (Livak and Schmittgen, 2001). For the pri-miR profiling, cDNA was diluted four times before qRT-PCR analysis with SYBR Green (Applied Biosystems) on a 384-well 7900HT Real-Time PCR (Applied Biosystems), using *PP2A* (At1g13320), *At2g283904*; *At5g15710*, and *UBQ10* (At4g05320) as reference genes (Czechowski *et al*., 2005). For the validation of pri-miR profiling results, qRT-PCR was performed with SYBR Green (Invitrogen) on a 96-well 7500 Fast Real-Time PCR (Applied Biosystems), using *PP2A* (At1g13320) as the reference gene. Differences in gene expression were considered significant for fold-changes ≥|1.5| between treated and control samples and for *P* < 0.05 according to two-tailed Student’s t-test. Mature miRNA quantification was conducted as described by Varkonyi-Gasic *et al*. (2007), using *PP2A* for normalization. The sequences of the qRT-PCR primers are given in Supplementary Table S1 (available at *JXB* online).

### High-throughput analysis of short-term responses to glucose

*Arabidopsis* Col-0 growth conditions in liquid MS/2, treatments with 2% glucose and 2% mannitol (osmotic control) for 4h, and RNA extraction protocol for the analysis of short-term glucose responses were performed as described previously (Matiolli *et al*., 2011). Preparation and deep sequencing of small RNA (sRNA) libraries was conducted according to the manufacturer’s instructions by Ambry Genetics (Aliso Viejo, CA, USA) using Illumina’s Genome Analyzer IIx. The reads were analysed using CLC Genomics Workbench software (CLC Bio). All reads were matched against *Arabidopsis* miRNA mature sequences downloaded from miRBase (release 18; Kozomara and Griffiths-Jones, 2011). Mismatches were not allowed in the matching and maximum number of hits for a read was one. The read count values were estimated as the reporting expression value for each treatment. Differential expression of miRNAs was detected by test on proportions and Kal’s Z-test. Small RNA library data from this study were deposited in the GEO database under the accession number GSE43546.

## Results

### *ago1-25*, *dcl1-11*, and *hyl1-2* mutants exhibit a glucose hyposensitive growth phenotype from germination to seedling establishment

**Fig. 1.**
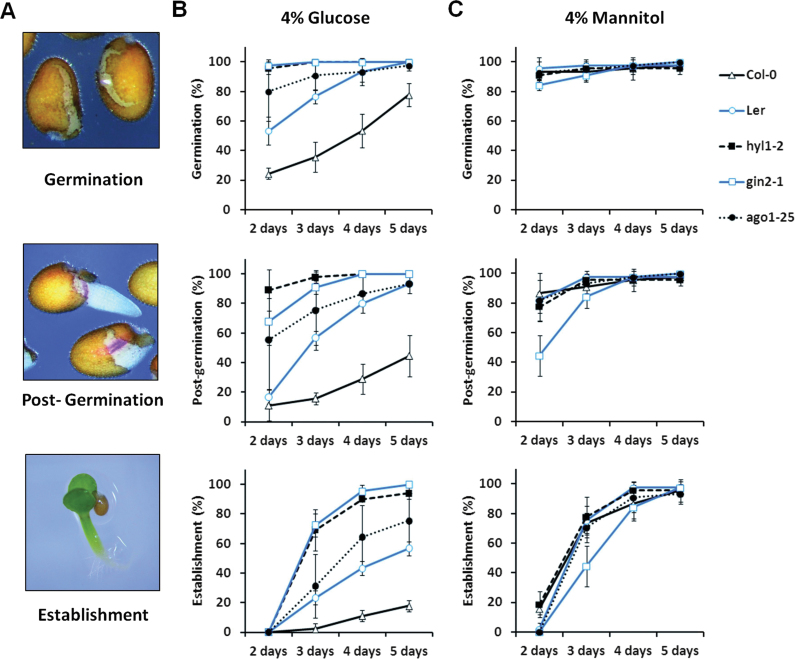
Glucose-induced delay of early seedling development is dependent upon miRNA machinery activity. (A) Developmental phases that were monitored; germination (testa rupture), post-germination (radicle emergence and elongation), and establishment (cotyledon expansion and greening). (B) Effects of 4% glucose on germination and development of miRNA-deficient mutants *ago1-25* and *hyl1-2* was less severe than for wild-type Col-0 and L*er* and was similar to the glucose-insensitive *gin2-1*. (C) Osmotic-control 4% mannitol could not reproduce the delay observed for glucose. In media not supplied with sugar, all seeds reached post-germination stage at 2 d and establishment within 3 d of light exposure (Supplementary Fig. S2A). In glucose-supplied media, growth arrest was not observed before seedling establishment stage was reached. Seeds of each genotype were sown in MS/2 plates supplied or not with the indicated sugar, kept for 2 d at 4 °C in the dark for stratification and transferred to continuous light (50 µmol m^–2^ s^–1^) at 24 °C. Germination, post-germination, and establishment were scored from 2–5 d of light exposure. Results presented are mean±SD of three independent experiments, each of which including 20 seeds (this figure is available in colour at *JXB* online).

In order to evaluate the involvement of miRNA-related regulatory pathways in glucose-induced delay of *Arabidopsis* early seedling development (Nonogaki *et al*., 2010; Eveland and Jackson, 2012), the miRNA-biogenesis mutants *dcl1-11* and *hyl1-2*, the miRNA-activity mutant *ago1-25*, and the corresponding wild type Col-0 were scored for germination (i.e. testa rupture), post-germination (i.e. radicle emergence and elongation), and establishment (i.e. cotyledon expansion and greening; Fig. 1A, Supplementary Fig. S1) in response to a range of glucose or mannitol (osmotic control) concentrations. The glucose-insensitive mutants *gin2-1* (HXK1 glucose-sensor knockout mutant in L*er* background; Moore *et al*., 2003) and *abi4-1*/*gin6* (Apetala2-type transcription factor involved in ABA signalling in Col-0 background; León and Sheen, 2003) were included as controls.

A first set of experiments using a range of glucose concentrations between 0 and 6% revealed that from germination to seedling establishment, *hyl1-2* and *ago1-25* were significantly less sensitive to high concentrations of glucose than Col-0 wild-type seedlings. Their levels of sensitivity were comparable to the glucose-insensitive mutant *abi4-1* (Supplementary Fig. S1). No differences in the rate of development between the genotypes were observed for seedlings grown on control media or media supplied with 1% glucose (Supplementary Fig. S1). Nevertheless, after 14 d of light exposure in the presence of 4% glucose, all seedlings of all genotypes reached the establishment phase (cotyledon expansion and greening; data not shown). Similar results were obtained with different seed batches (data not shown).

From these initial data, it was deduced that 4% glucose is a suitable concentration to accurately evaluate the degree of glucose sensitivity of *hyl1-2* and *ago1-25* (Supplementary Fig. S1). Under this condition and after 2 d of light exposure, only 50% of L*er* and 20% of Col-0 seedlings germinated (Fig. 1B), while 80% of *ago1-25* and nearly all *hyl1-2* seeds germinated (Fig. 1B). This result indicates a significant lower sensitivity to 4% glucose of the two miRNA-deficient genotypes as compared to the wild type. Moreover, their level of glucose hyposensitivity was comparable to the one exhibited by *gin2-1* (Fig. 1B). The same differences in glucose sensitivity were essentially maintained among the different genotypes for both the post-germination and seedling establishment stages until after 5 d of light exposure (Fig. 1B). The glucose hyposensitivity of the mutants was not due to differential capacity to germinate since in the absence of glucose, early seedling development was indistinguishable between genotypes (Supplementary Fig. S2). In addition, 4% mannitol (i.e. the osmotic control) was unable to reproduce the effects of 4% glucose (Fig. 1C, Supplementary Fig. S2A), indicating that the glucose-mediated delay of seedling development is not due to osmotic stress. As a means to further support this set of results, glucose sensitivity of the relatively weak mutant allele, *dcl1-11*, was also evaluated. As expected, it was found to be hyposensitive to 4% glucose (Supplementary Figs S2B and S3). Together, these observations imply that a miRNA-related machinery is involved in glucose-induced development delay from germination to seedling establishment.

Since the effect of glucose can be mediated by the ABA signalling pathway during early seedling development (León and Sheen, 2003; Dekkers *et al*., 2008), it was important to verify whether seedling development in the miRNA-deficient mutants would also be hyposensitive to ABA (Supplementary Fig. S4). As a control, the *abi4-1* mutant was shown to be ABA hyposensitive (Supplementary Figs S2C and S4; Finkelstein, 1994). While *ago1-25* and *hyl1-2* were found to be more sensitive to ABA than the wild type from germination to establishment, hypersensitivity of *dcl1-11* was evident in the post-germination and establishment stages (Supplementary Figs S2C and S4), as previously described (Lu and Fedoroff, 2000; Kim *et al*., 2008; Zhang *et al*., 2008; Earley *et al*., 2010). The data suggest that the glucose hyposensitivity phenotype of *ago1-25*, *dcl1-11* and *hyl1-2* is at least partly independent on ABA-related signalling pathway.

### Expression of miRNA genes in response to glucose

**Fig. 2.**
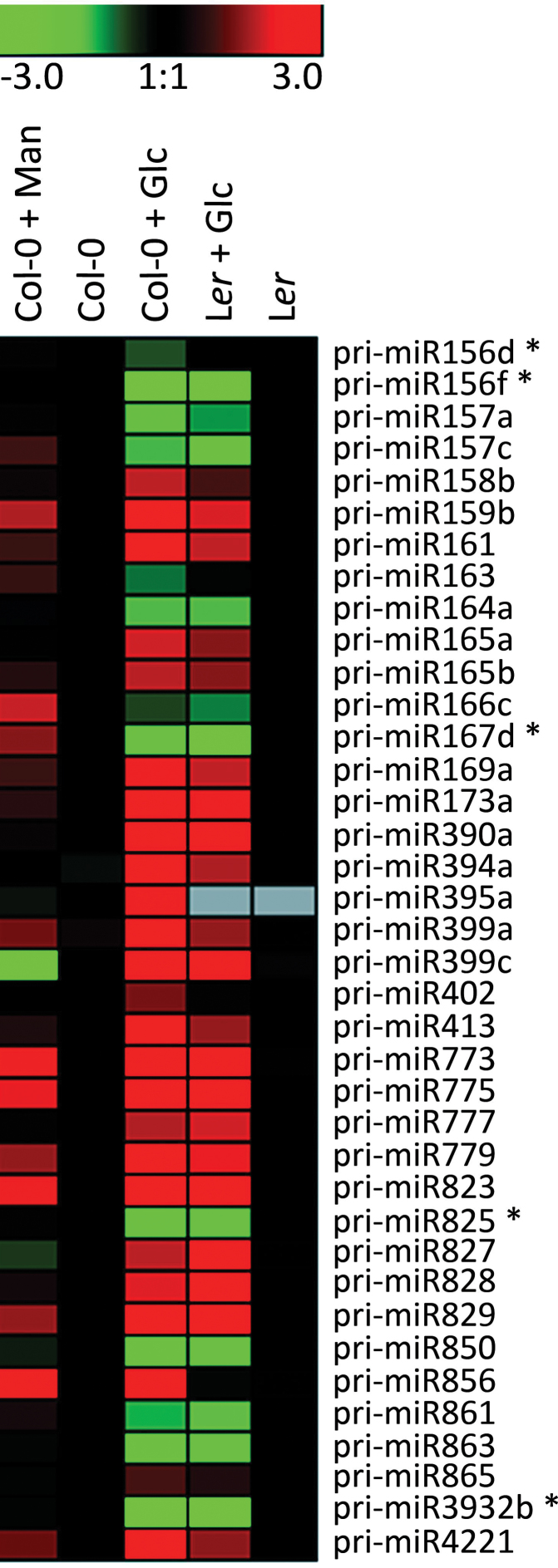
pri-miR levels in Col-0 and L*er* seedlings after 3 d of light exposure grown in control media, 4% glucose (Glc) media, and 4% mannitol (Man) media. Potential HXK1-sensing dependent pri-miRs are marked with an asterisk. Seed dormancy was previously broken by imbibition for 2 d at 4° C in the dark. This set of glucose-responsive pri-miRs (column 3) presented at least 1.5-fold expression difference to non-treated control samples (column 2), at least 2-fold difference to mannitol-treated samples (column 1), and were considered to be significantly different according to Student’s t-test (*P* < 0.05). The colours represent the log_2_ values of the relative quantification between (Col-0 vs. Col-0 + treatment) and (L*er* vs. L*er* + treatment); green indicates repression, red indicates induction. Mannitol-treated L*er* is not shown.

To identify which miRNAs may be involved in the glucose-induced early seedling development delay, a platform established for quantification of 200 pri-miR transcripts by qRT-PCR was used (Pant *et al*., 2009). Although pri-miRs are not the biologically active molecules, their abundances often reflect the levels of the mature miRNA (Pant *et al*., 2009). While this approach allows for the verification of expression changes for a specific *MIRNA* locus, special assays are required to quantify the mature miRNAs. These assays, however, cannot always distinguish mature miRNA species of the same miRNA family due to their extensive sequence homology. The expression analysis was carried out with RNAs from Col-0, L*er*, and *gin2-1* seedlings grown for 3 d in light in media with or without 4% glucose or mannitol (osmotic control). A total of 38 pri-miRs belonging to 32 families were found to be differentially expressed in the presence of 4% glucose as compared to the control in Col-0. Of these, 33 showed the same response in L*er* accession (Fig. 2, Supplementary Table S2). Since the pri-miR platform was designed based on Col-0 background, primer mismatches may explain the differences observed between Col-0 and L*er* profiles. In addition, five of the 38 glucose-responsive miRNA precursors—pri-miR156d, pri-miR156f, pri-miR167d, pri-miR3932b, and pri-miR825—were found to require the glucose-sensing activity of HXK1, as revealed by the comparison between pri-miR accumulation in *gin2-1* and L*er* seedlings. pri-miR172, which is a downstream component of a regulatory cascade that involves miR156 (Martin *et al*., 2010; Nonogaki, 2010) was found to be upregulated, yet it did not pass the threshold to be considered significant. Quite noticeably, the mature miRNAs derived from several of the identified glucose-responsive pri-miRs are known to target mRNAs that participate directly or indirectly in different aspects of plant development (Table 1, Supplementary Table S2).

**Table 1.** Long-term 4% glucose-regulated pri-miRs related to development control

	Relative quantification by qRT-PCR					
pri-miRNA	4% Glucose	4% Mannitol	Development	Target gene family	Developmental process	Reference
pri-miR156d^†^	0.25±0.05^a^	0.76±0.10^b^	0.18±0.05	SPL	Embryo development/ vegetative leaves emergence	Wu *et al*. (2009); Nodine and Bartel (2010)
pri-miR156f^†^	0.11±0.11^a^	0.80±0.26^b^	nd			
pri-miR159b^†^	5.26±0.24^a^	2.14±0.09^b^	2.10±0.47^c^	MYB	Seedling growth and development/ABA response	Reyes and Chua (2007)
pri-miR166c^†^	0.27±0.02^a^	2.12±0.29^b^	0.08±0.03^c^	HD-ZIP III	Leaf flattening	Husbands *et al*. (2009)
pri-miR169a^†^	2.05±0.28^a^	1.18±0.26^b^	1.33±0.19^c^	NF-Y	Embryo development/ ABA response	Li *et al*. (2008); Yamamoto *et al*. (2009)
pri-miR390a^†^	4.00±0.92^a^	1.59±0.65^b^	2.52±0.24^c^	TAS3	Lateral root development/l eaf polarity	Marin *et al*. (2010)
pri-miR773	19.81±5.74^a^	3.87±1.33^b^	3.93±0.52^c^	MET2	Biotic stress response	Fahlgren *et al*. (2006); Li *et al*. (2010)
pri-miR775	21.76±3.54^a^	3.98±0.86^b^	9.80±1.84^c^	GT	Arabinogalactan- protein biosynthesis	TarBase: Vergoulis et al. (2012)
pri-miR823	18.98±1.71^a^	4.24±2.17^b^	5.24±1.14^c^	CMT3	Embryo development	TarBase: Vergoulis et al. (2012)
pri-miR828	8.44±1.19^a^	5.07±0.85^b^	2.04±0.51^c^	MYB	Nutrient availability control	Luo *et al*. (2012)

Relative quantification values for glucose and mannitol are from samples grown in media supplied with 4% of the respective sugar in comparison with the untreated control, all grown for 3 d in light; relative quantification values for development are from samples grown for 1 d in light compared with samples grown for 3 d in light, both under control conditions. All samples are Col-0; values are mean ± SD of at least three biological replicates. Target gene families are based on the indicated published results or according to TarBase database (Vergoulis *et al.*, 2012). ^†^Pri-miRs that had the correspondent miRNA/miRNA family sequence identified as responsive to short-term (i.e. 4h) 2% glucose, based on deep sequencing analysis (Supplementary Table S3); ^a–c^Changes in transcript accumulation were considered significant for differences with fold-change ≥|1.5| and according to Student’s t-test (*P* < 0.05): ^a^glucose-treated vs. untreated; ^b^mannitol-treated vs. glucose-treated; ^c^development vs. glucose-treated samples; nd, not detected.

The differential expression induced by glucose was confirmed by qRT-PCR for 10 of the 12 development-related pri-miRs that were evaluated (83%; Table 1), essentially validating the high-throughput pri-miR quantification data. Since 4% glucose delays seedling development, it was also necessary to distinguish true glucose effect from developmental cues that may influence pri-miR expression. To this end, the expression of the 10 validated pri-miRs was compared between wild-type seedlings grown in control conditions for 1 d in light, which corresponds to the developmental stage reached by seedlings grown for 3 d in light in presence of 4% glucose (~30% germination; Fig. 1), and seedlings grown for 3 d in light in media not supplied with glucose, which stands for the control conditions used for the pri-miR profiling (100% germination; Fig. 2, Supplementary Table S2). All pri-miRs tested except pri-miR156d showed significant differences between glucose- and development-promoted changes (Table 1). Thus, for these nine pri-miR, developmental signals may contribute to no more than half of the expression changes observed in 4% glucose-grown seedlings (Table 1). pri-miR156f could not be detected in seedlings grown for 1 d in light, indicating a very low expression level, while 4% glucose repressed its expression by 9-fold (Table 1).

To further support genuine glucose-mediated responses, changes of pri-miR expression promoted by short-term 4% glucose treatments were evaluated in Col-0. pri-miR expression alterations resulting from treatment with 4% glucose applied for 4h on seedlings grown for 3 d in light (Supplementary Fig. S5) followed the same expression trends as those observed for seedlings grown for 3 d in light in 4% glucose media (Table 1). The only exception was pri-miR169a (Supplementary Fig. S5), which presented an opposite response that may reflect an alternative regulation pattern between short- and long-term responses. These data, therefore, essentially corroborate the notion of true glucose-promoted responses. The same tendency of glucose-promoted responses between short- and long-term treatments was also observed for the target genes (data not shown).

**Fig. 3.**
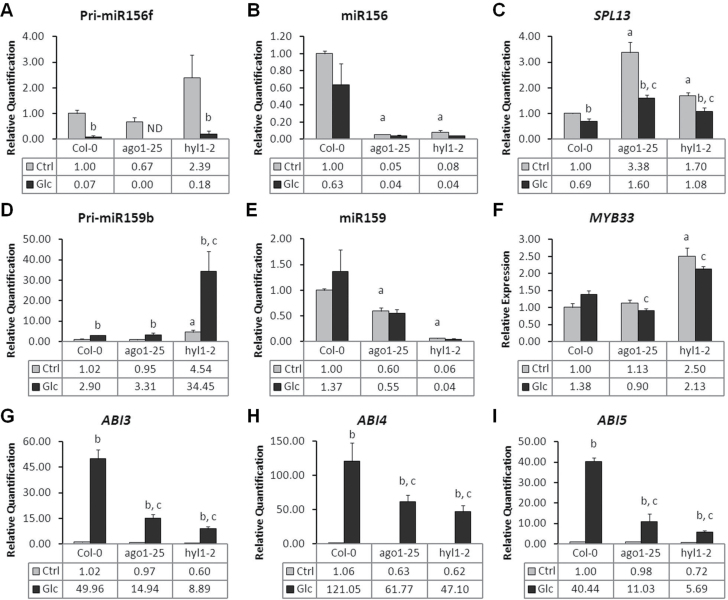
Expression of (A) pri-miR156f, (B) miR156, (C) *SPL13*, (D) pri-miR159b, (E) miR159, (F) *MYB33*, (G) *ABI3*, (H) *ABI4*, and (I) *ABI5* in Col-0, *ago1-25* and *hyl1-2* seedlings grown in 4% glucose or control media after 3 d of light exposure. All expression values are in comparison to untreated Col-0. Values are mean±SD of three biological replicates. Below each graph, the relative transcript abundance is given. Changes in transcript accumulation were considered significant for differences with fold-change ≥|1.5| and according to Student’s t-test (*P* < 0.05) for the following comparisons: a, untreated *ago1-25* or *hyl1-2* vs. untreated Col-0; b, glucose-treated vs. untreated samples (same genotype); c, glucose-treated mutant vs. glucose-treated Col-0. nd, not detected.

The subset of nine pri-miRs whose expression was specifically affected by 4% glucose and could not be attributed to developmental cues (Table 1) was used to evaluate the assumption that pri-miR accumulation may reflect the levels of the corresponding biologically active mature miRNA (Pant *et al*., 2009). The mature miRNA of the families corresponding to the validated pri-miRs were quantified in Col-0, *ago1-25* and *hyl1-2* seedlings grown for 3 d in light in the presence and absence of 4% glucose. This analysis revealed that the pattern of mature miRNA accumulation follows the trend of pri-miR expression changes in response to glucose (Fig. 3, Supplementary Fig. S6). However, an opposite pattern was observed for pri-miR169a/miR169, which may explain the increase of the *NF-YA5* target transcript in response to glucose, indicating that glucose may also have effects on miRNA maturation (Supplementary Fig. S6B). As expected, a clear dependence on HYL1 activity was seen for the production of all miRNAs evaluated. AGO1 also appeared to be required for proper accumulation of miR156, miR159, miR166, miR169, miR773, miR775, and miR823 (Fig. 3, Supplementary Fig. S6), which is in agreement with previous reports (Vaucheret *et al*., 2004; Tang *et al*., 2012). These data suggest that pri-miR level indeed reflect the abundance of the corresponding miRNA and that glucose can influence the pool of miRNA species.

Finally, glucose-promoted changes in miRNA accumulation were evaluated during a short-term (i.e. 4h) treatment with a reduced sugar concentration (i.e. 2%), which is known not to induce ABA accumulation (Matiolli *et al*., 2011). Based on deep sequencing of *Arabidopsis* Col-0 sRNAs, 29 known unique miRNA sequences out of 155 detected in seedlings grown in liquid MS/2 media for 6 d in light were found to be altered by the short-term treatment with 2% glucose (Supplementary Table 3; Table 1). This result further supports the notion that miRNA accumulation can be regulated by glucose. A comparison of these short-term, glucose-mediated miRNA profile alterations with the pri-miR/miRNA changes detected in seedlings grown for 3 d in light in the presence of 4% glucose (i.e., long-term treatment; Fig. 2, Supplementary Table S2) revealed a common set of 12 miRNAs, six of them showing similar responses to glucose, while the other six exhibited opposite responses (Supplementary Table 3; Table 1). Such differences may be due to differences in developmental stages or glucose concentration between the experiments, but also suggest that short- and long-term glucose-mediated regulation on miRNA expression can be different.

### mRNA misregulation resulting from miRNA-pathway deficiency may be associated with glucose hyposensitivity of *ago1-25*, *dcl1-11*, and *hyl1-2* from germination to seedling establishment

In an attempt to get insights into how miRNA-related regulatory pathways may mediate sensitivity to glucose during early seedling development, the set of nine validated glucose-responsive pri-miRs (Table 1), whose mature forms are known to be involved in developmental cues (miR156, miR159, miR166, miR169, miR390, miR823), biotic stress response (miR773), adaptation to mineral nutrient availability (miR828), and arabinogalactan-protein biosynthesis (miR775), were analysed in more detail. The analysis consisted of evaluating the long-term glucose-mediated expression changes of these nine selected *MIRNA* genes together with their respective target genes in the Col-0, *ago1-25*, and *hyl1-2* mutant backgrounds.

Deficiencies in HYL1 or AGO1 were shown to differently affect the accumulation of the nine selected pri-miRs (Fig. 3, Supplementary Fig. S6). Since HYL1 is required for pri-miR processing (Szarzynska *et al*., 2009; Voinnet, 2009), higher accumulation of pri-miRs in *hyl1-2* would be expected. All but one (pri-miR166c) of the nine pri-miRs tested were found to be more abundant (pri-miR156f, pri-miR159b, pri-miR169a, pri-miR390a, pri-miR773) or present at equal levels (pri-miR775, pri-miR823, pri-miR828) in *hyl1-2* as compared to the wild type, in the absence and presence of glucose (Fig. 3, Supplementary Fig. S6). pri-miR166c was found to be significantly reduced in *hyl1-2*, indicating the existence of an alternative role for HYL1 or a feedback regulation affecting pri-miR166c expression (Supplementary Fig. S6).

In untreated *ago1-25* plants, the accumulation of most (seven out of nine) of the pri-miRs was not affected when compared to the wild type, a result that was expected since AGO1 is assumed not to be directly involved in miRNA biogenesis (Fig. 3, Supplementary Fig. S6). However, pri-miR169a and pri-miR773 levels were reduced in *ago1-25*, possibly reflecting an indirect effect, such as feedback regulation (Supplementary Fig. S6). Most glucose-induced pri-miR accumulation in *ago1-25* appears to be attenuated in comparison with Col-0. In contrast, both the pri-miRs that are repressed by glucose, pri-miR156f and pri-miR166c, responded more strongly in *ago1-25* in comparison to Col-0 (Fig. 3, Supplementary Fig. S6). The reasons for these opposite responses are unclear, but could possibly derive from altered gene expression programmes due to AGO1 deficiency (Tang *et al*., 2012).

An inverse correlation between changes in pri-miR levels and the accumulation of the respective target mRNA in response to glucose in the wild type was observed for pri-miR166c/*PHV*, pri-miR390/*TAS3*, pri-miR775/*At1g53290*, and pri-miR773/*MET2* (Supplementary Fig. S6). For the remaining five pri-miRs and their targets (pri-miR828/*TAS4*, pri-miR823/*CMT3*, pri-miR169a/*NF-YA5*, pri-miR156f/*SPL13*, and pri-miR159/*MYB33*), the glucose-induced modulation of pri-miR levels was correlated with parallel changes of their targets (Fig. 3, Supplementary Fig. S6). Overall, the wild-type pattern of target mRNA accumulation in control conditions and/or in response to glucose was found to be qualitatively similar in the *ago1-25* and *hyl1-2* mutants (Fig. 3, Supplementary Fig. S6). For *PAP1* mRNA accumulation, which is target of *TAS4*-derived *trans*-acting small interfering RNA (ta-siRNA) as part of a feedback regulatory loop (Luo *et al*., 2012), quantitative differences were observed in both mutants. Accumulation of mRNA was also deregulated in comparison to the wild type for *TAS4* and *MYB33* in *hyl1-2* and for *NF-YA5* and *SPL13* in *ago1-25* (Fig. 3, Supplementary Fig. S6). Additionally, the mRNA level of the transcriptional factor *SPL13*, whose accumulation was reported to lead to a delay in the emergence of vegetative leaves (Martin *et al*., 2010; Nonogaki, 2010), was found to be significantly higher in both *ago1-25* and *hyl1-2* as compared to Col-0 (Fig. 3C).

Short-term responses promoted by 4% glucose on pri-miR expression in Col-0 were found to be basically the same as long-term responses (Fig. 3, Supplementary Figs S5 and S6). The same trend was also found for *ago1-25* and *hyl1-2* (Fig. 3, Supplementary Figs S5 and S6). In addition, most responses to glucose in *dcl1-11* follow the responses observed in *hyl1-2* (Supplementary Fig. S5). However, in control conditions, six pri-miRs (pri-miR159b, pri-miR166c, pri-miR169a, pri-miR775, pri-miR823 and pri-miR828) were present at lower levels in *dcl1-11* than in Col-0 (Supplementary Fig. S5). It is unclear however what causes the reduction of these pri-miR levels in *dcl1-11*, but could be related to this specific *dcl1* allele.

How these observed quantitative differences in mRNA levels of these miRNA target genes in the miRNA-deficient mutants are involved in their glucose hyposensitive phenotype remains elusive. A potential candidate to explain the glucose hyposensitive phenotype of *ago1-25* is the transcription factor gene *MYB33* which is regulated by miR159b and mediates ABA-induced inhibition of seedling development (Reyes and Chua, 2007). The lower level of *MYB33* mRNA in *ago1-25* after long-term treatment with 4% glucose compared with the wild type may partly explain the attenuated development delay of the mutant in such conditions (Fig. 3F). However, the higher amount of *MYB33* mRNA in response to glucose in *hyl1-2* in comparison with the wild type would be expected to result in glucose-promoted development delay, which is the opposite of the observed glucose hyposensitive phenotype of *hyl1-2* (Fig. 3F). MYB33 is part of an ABA-related network which involves ABI3 and ABI5 and retards seedling development under abiotic stress conditions (Reyes and Chua, 2007). Mutants for ABI3, ABI4, and ABI5 have been described as *gin* mutants (reviewed in Gibson, 2005), so it was important to evaluate the glucose-induced modulation of the expression of these three genes in the miRNA-pathway mutants. *ABI3*, *ABI4*, and *ABI5* expression were found to be strongly induced by 4% glucose during long-term treatment in Col-0 seedlings grown for 3 d in light (Fig. 3G–I) but not by mannitol, discounting the possibility of an osmotic effect (Supplementary Fig. S7A). Moreover, the fold-change observed for *ABI3*, *ABI4*, and *ABI5* in 4% glucose-grown seedlings could only partially be attributed to developmental signals, as significantly different responses were observed between seedlings 1 and 3 d after light exposure (Supplementary Fig. S7B). This glucose-specific effect was significantly attenuated in the *ago1-25* and *hyl1-2* mutants (Fig. 3G–I). This result suggests that while *ABI3*, *ABI4*, and *ABI5* are not known to be direct miRNA targets, it is possible that the lower levels of the mRNAs of these three ABA signalling elements are, at least partly, responsible for the glucose hyposensitive phenotype of miRNA-pathway mutants. The observed lower accumulation of *ABI3*, *ABI4*, and *ABI5* mRNAs in *ago1-25* and *hyl1-2* could be explained by an unknown upstream transcriptional activator that is target of a glucose-dependent miRNA.

## Discussion

Developmental processes and responses to environmental changes may rely on fast and fine adjustments of mRNA or protein profiles, which can be partially achieved through miRNA-mediated control of mRNA decay and/or translation (Voinnet, 2009; Floris *et al*., 2009). Additionally, miRNAs in plants have been proposed to control developmental transitions by preventing premature mRNA expression (Nodine and Bartel, 2010). Thus, the regulation of *MIRNA* gene expression is crucial for proper growth and development. The modulation of the expression of up to 38 pri-miR genes by long-term treatment with glucose shown in this study, mainly via an HXK1-sensing independent pathway, reveals a new aspect of how sugar signalling could impact on seedling growth, development, and physiology through miRNA-related regulatory schemes (Fig. 2, Supplementary Table S2). This notion is in agreement with previous reports (Yang *et al*., 2013) and was further supported by the observation that the pattern of glucose-mediated regulation of seven among eight tested pri-miRs reflects the profile of the corresponding mature miRNAs (Fig. 3, Supplementary Fig. S6). Only one case of inverse correlation was observed for pri-miR169a/miR169 (Supplementary Fig. S6), which might reflect antagonist transcriptional and post-transcriptional control (Ramachandran and Chen, 2008; Viswanathan *et al*., 2008; Ren *et al*., 2012; Manavella *et al*., 2012).

In wild-type seedlings, the consequences of pri-miR regulation by glucose on the accumulation of their target mRNA was not found to be fully predictable based solely on the activity of miRNA-mediated mRNA degradation. Among nine target mRNAs that were analysed in more detail, four showed an inverse correlation between the levels of glucose-promoted changes of pri-miR levels and of the mRNA in the wild type (Fig. 3, Supplementary Fig. S6). The trend observed for the remaining five target mRNAs was to follow the expression pattern of the pri-miR (Fig. 3, Supplementary Fig. S6). Such a finding could reflect control of miRNA activity, which seems to occur despite its accumulation and may be related to the developmental stage (Alonso-Peral *et al*., 2012). Feedforward (Vidal *et al*., 2010), dampening regulatory mechanisms or differential tissue-specific expression patterns of the miRNA and its target (Voinnet, 2009) are other possible explanations. For example, miR390 and *TAS3* are part of a ta-siRNA regulatory loop and are known to be specifically expressed at sites of lateral root initiation (Marin *et al*., 2010), implying that changes in *ARF3* levels promoted by *TAS3*-derived ta-siRNA may only be detectable locally (Supplementary Fig. S6C).

In comparison to the wild type, *ago1-25* and *hyl1-2* mutants had variable consequences on target mRNA accumulation in both control and 4% glucose conditions (Fig. 3, Supplementary Fig. S6). Although target mRNA would be expected to accumulate to higher levels in miRNA mutants, it was found to be true only for a limited set of targets in *ago1-25* and/or *hyl1-2* either in control conditions and/or in response to glucose, (*NF-YA5*, *TAS3*, *At1g53290*, *MYB33*, and *SPL13*; Fig. 3, Supplementary Fig. S6). The absence of systematic increase of target mRNA levels in these mutants may be due to different reasons, including the strength of the mutation, which is leaky in the case of *ago1-25*, and redundancy, which has been reported for AGO proteins (Mi *et al*., 2008) and HYL1 (Vazquez *et al*., 2004; Szarzynska *et al*., 2009).

The few differences in target gene mRNA levels that were observed in *ago1-25* and *hyl1-2* as compared to the wild type did not provide a clear-cut reason for the glucose hyposensitive phenotype of these mutants. For instance, *MYB33* may partly be involved in the *ago1-25* glucose hyposensitive phenotype but cannot be responsible for the reduced sensitivity to glucose in *hyl1-2* (Fig. 3F). It is, however, possible that rather than a single causal change it is the combination of small or subtle alterations that underlies the glucose hyposensitive phenotype of the miRNA-deficient mutants. For instance, *dcl1* embryo patterning defects cannot be phenocopied solely by miR156-resistant *SPL10* and *SPL11* transgenes, despite their great derepression in *dcl1* (Nodine and Bartel, 2010). In addition, besides their activity in target mRNA cleavage, translational inhibition mediated by miRNA may also have an important functional role (Brodersen *et al*., 2008; Yang *et al*., 2012). Alternatively, since ABI3, ABI4, and ABI5 are known to modulate sensitivity of seedling development to glucose (Arenas-Huertero *et al*., 2000; Cheng *et al*., 2002; Arroyo *et al*., 2003; Dekkers *et al*., 2008), the weaker accumulation of *ABI3*, *ABI4*, and *ABI5* mRNA in response to long-term treatment with glucose in *ago1-25* and *hyl1-2* mutants could also be directly involved in the glucose hyposensitive phenotype of these mutants during this developmental window (Fig. 3G–I). How the glucose-mediated regulation of these ABA signalling elements is altered in the miRNA-deficient mutants is unknown but is likely to be indirectly related to inappropriate expression of developmental programmes.

To conclude, the regulation of miRNA expression by glucose supports the notion that control of mRNA decay and/or translation represents another mechanistic aspect involved in tuning the glucose regulatory network. A key issue now is to identify the gene expression programmes related to glucose-promoted developmental responses.

## Supplementary material

Supplementary data are available at *JXB* online.

Supplementary Table S1. Primer sequences.

Supplementary Table S2. Glucose-regulated pri-miRs and corresponding miRNA targets.

Supplementary Table S3. Known miRNAs present in control and short-term 2% glucose-treated seedlings.

Supplementary Fig. S1. Effects of different concentrations of glucose on early seedling development of wild-type Col-0, miRNA-deficient mutants *ago1-25* and *hyl1-2*, and glucose-insensitive mutant *abi4-1/gin6*.

Supplementary Fig. S2. Statistical analyses of germination and development efficiencies.

Supplementary Fig. S3. Effects of glucose on early seedling development of Col-0, L*er*, *hyl1-2*, *gin2-1*, and *dcl1-11*.

Supplementary Fig. S4. Effects of ABA on early seedling development of Col-0, *aba2-1*, *abi4-1*, *ago1-25*, *dcl1-11*, and *hyl1-2*.

Supplementary Fig. S5. Short-term effects of 4% glucose on accumulation of *MIRNA* genes pri-miRs in Col-0, *ago1-25*, *dcl1-11*, and *hyl1-2* seedlings after 3 d of light exposure.

Supplementary Fig. S6. Long-term effects of glucose on accumulation of *MIRNA* genes pri-miR, their corresponding miRNA family and respective targets in Col-0, *ago1-25* and *hyl1-2* seedlings grown in 4% glucose or control media after 3 d of light exposure.

Supplementary Fig. S7. Validation of glucose-promoted changes on *ABI3*, *ABI4* and *ABI5* accumulation.

Supplementary Data
