# Supplementary material for: Involvement of microRNA-related regulatory pathways in the glucose-mediated control of Arabidopsis early seedling development
Source: J Exp Bot. 2013 Aug 30;64(14):4301–12. doi: 10.1093/jxb/ert239 (PMC3808316; doi:10.1093/jxb/ert239)
Supplement: Supplementary Data [file supp_64_14_4301__index.html]

Involvement of microRNA-related regulatory pathways in the glucose-mediated control of Arabidopsis early seedling development — Involvement of microRNA-related regulatory pathways in the glucose-mediated control of Arabidopsis early seedling development — Supplementary Data 

# Involvement of microRNA-related regulatory pathways in the glucose-mediated control of *Arabidopsis* early seedling development

## 

Data files

**Files in this Data Supplement:**

- Supplementary Data - Supplementary Data
- Supplementary Data - Supplementary Data
