# Supplementary material for: Involvement of microRNA-related regulatory pathways in the glucose-mediated control of Arabidopsis early seedling development
Source: J Exp Bot. 2013 Aug 30;64(14):4301–12. doi: 10.1093/jxb/ert239 (PMC3808316; doi:10.1093/jxb/ert239)
Supplement: Supplementary Data [file supp_ert239_jexbot096099_file002.pdf]

Journal of Experimental Botany, Involvement of microRNA-related regulatory pathways in the glucose-mediated control of Arabidopsis early seedling development.

Gustavo Turqueto Duarte, Cleverson Carlos Matiolli, Bikram Datt Pant, Armin Schlereth, Wolf-Rüdiger Scheible, Mark Stitt, Renato Vicentini, Michel Vincentz

Supplementary Table S1.

		Primer Sequences		
**Target**	**AGI**	**Forward (5' - 3')**	**Reverse (5'- 3')**	**Reference**
*PAP1/AtMYB75**	AT1G56650	GGTTGAACTATTTGAAGCCA	GCAATTAAAGACCACCTATTC	Luo *et al.*, 2011
TAS4	AT3G25795	CGACCTCGATCCTTCACCT	ATTTTCTAGACCTGCATTGTTTAT	
AT2G28390	AT2G28390	AACTCTATGCAGCATTTGATCCACT	TGATTGCATATCTTTATCGCCATC	Pant *et al.*, 2009
AT5G15710	AT5G15710	AAGTTCGCCAGCAAAACATGTC	GCTGACCAGGTCTTCATTAACACTG	
PP2A/PDF2	At1g13320	TAGATCGCTCGGAACTTGGAAA	CCTCACCAAAACTCAAATCACTCC	
Pri-miR156d	AT5G10945	CAGAAGAGAGTGAGCACACAAAGGG	GTGAGCACGCAAAAGCAACCATATAC	
Pri-miR156f	AT5G26147	TGGTGAGGAATTGATGGTGACA	CCTTCAAATATGCAAGAAAGCCAC	
Pri-miR159b	AT1G18075	GGAGGGTTTAGCAGGGTGAAGTAAAG	CCAAAGAAGAGTGAAGCCATTAAAGGG	
Pri-miR166c	AT5G08712	AGTGTTGAGAGGATTGTTGTCTGGC	GAATGAAGCCTGGTCCGAGAATCATC	
Pri-miR169a	AT3G13405	AAAGTAACATGATCGGCAAGTTGTCC	GCGACACAAAGTAACGTGTAGCC	
Pri-miR390a	AT2G38325	TAGCGCCATGATGATCACATTC	AATGAAACTCAGGATGGATAGCG	
Pri-miR399c	AT5G62162	CATCTTTCTATTGGCAGGCGACTTGG	AAGCAGTGACAGGGCAACTCTCC	
Pri-miR773	AT1G35501	AGGAGGCAATAGCTTGAGCAAA	AGGTGACAGCTTTAGTCGATGGA	
Pri-miR775	AT1G78206	CATTGAAACTGTCTTTCAACATTCCA	TGGCACTGCTAGACATCGAAAAT	
Pri-miR823	AT3G13724	CCATTTAGTTCTAGTGGGTGGTGAT	GATATGTTTCACTGTTACCATTACCAATCT	
Pri-miR827	AT3G59884	TCCTTGTGTTGATCGATTGGTTTA	CGATGCAAAACCACGAAAGAG	
Pri-miR828	AT4G27765	AAATGATTCACTCACTCGTAT	GATATTAAATAGTCCCACTTCC	
UBQ10	AT4G05320	GGCCTTGTATAATCCCTGATGAATAAG	AAAGAGATAACAGGAACGGAAACATAGT	
*ABI3**	AT3G24650	GCAGTGCCGCCTCAATTAC	TTCTGGTTTCCATCCCTGCC	This Work
ABI4	AT2G40220	GAGGTGGCGTTAGGGCAGG	GGTGGATGAGTTATTGATAGAC	
*ABI5**	AT2G36270	GCAAGAAAACAAGCATATACAG	TTCCTCTTCCTCTCCAACTC	
*ARF3**	AT2G33860	CTGTCTCTGAGGGGATTCG	GGCTCCACCATCCGAACAAG	
*AT1G53290**	AT1G53290	TATCGAAGAGGAGTACAGTAAG	TAGCAGAGAGAGTCGATCTG	
*CMT3**	AT1G69770	TCAGGTTCACAATCAAAGTCC	AATTCGCTCCCTTTCTCTTGG	
*MET2/DMT2**	AT4G14140	AGGTTTACGCTATGATGCTGG	GTAGTTCCATACTCTTTGTTTAT	
*MYB33**	AT5G06100	GCACGTATGGCTGCACATTTG	CACTCAAGTGCCTCAACATGC	
*NF-YA5**	AT1G54160	AATGCCGTAAACCGTACCTTC	GCTTCTTTGTATTGAGGAAACG	
*PHV/ATHB9**	AT1G30490	AACATGAAGAGTTTCTCGAAG	AGCACAACCTCCAACCACATT	
*SPL13A**	AT5G50570	ACAATGCAGCAGGTTTCATG	GACGACCGATATGTTCAGGC	
TAS3	AT3G17185	AAACATAACCTCCGTGATGC	GCTCAGATAGGATAACACCG	

Note. Primer pairs spanning exon-exon junctions are marked with an asterisk.

Supplementary Table S1. Continued.

	miRNA Primer Sequences		
**miRNA**	**Amplification Forward (5' - 3')**	**Reverse Transcription Primer (5'- 3')**	**Reference**
miR159	CGGCGGTTTGGATTGAAGGGA	GTCGTATCCAGTGCAGGGTCCGAGGTATTCGCACTGGATACGACAAGAGC	This Work
miR166	CTCGCTTCGGACCAGGCTTCA	GTCGTATCCAGTGCAGGGTCCGAGGTATTCGCACTGGATACGACGGGGAA	
miR169	CGTGAGCAGCCAAGGATGACT	GTCGTATCCAGTGCAGGGTCCGAGGTATTCGCACTGGATACGACTCGGCA	
miR390	GGACGGAAGCTCAGGAGGGAT	GTCGTATCCAGTGCAGGGTCCGAGGTATTCGCACTGGATACGACGGCGCT	
miR773	CGATGCGTTTGCTTCCAGCTTT	GTCGTATCCAGTGCAGGGTCCGAGGTATTCGCACTGGATACGACGAGACA	
miR775	GCGGCGGTTCGATGTCTAGCA	GTCGTATCCAGTGCAGGGTCCGAGGTATTCGCACTGGATACGACTGGCAC	
miR823	GGACGGTGGGTGGTGATCATA	GTCGTATCCAGTGCAGGGTCCGAGGTATTCGCACTGGATACGACATCTTA	
miR828	CGGCGGTCTTGCTTAAATGAGT	GTCGTATCCAGTGCAGGGTCCGAGGTATTCGCACTGGATACGACTGGAAT	
miR156	GCGGCGGTGACAGAAGAGAGT	GTCGTATCCAGTGCAGGGTCCGAGGTATTCGCACTGGATACGACGTGCTC	Varkonyi-Gasic *et al.*, 2007
miRuniversal-R*	-	GTGCAGGGTCCGAGGT	

Note. The miRuniversal is the amplification reverse primer for all miRNAs.

Supplementary Table S2. Glucose-regulated pri-miRs and corresponding miRNAs targets.

miRNA Family	4% Glucose-responsive pri-miRNAs	4% Mannitol fold | 4% Glucose fold	Target Family	Target Genes
156/157	156d<br>156f<br>157a<br>157c	1.24 | -1.67<br>-1.02 | -7.62<br>1.24 | -2.83<br>1.95 | -2.50	SPL	AT1G27360, AT1G27370, AT1G53160, AT1G69170, AT2G33810*, AT2G42200, AT3G15270*, AT3G57920, AT5G43270, AT5G50570, AT5G50670
158	158b	1.34 | 3.42	PPR	-
159	159b	3.24 | 6.70	MYB	AT2G26950, AT2G32460, AT4G26930, AT5G55020
161	161a	1.89 | 4.90	PPR	AT1G06580, AT1G62590, AT1G62670, AT1G62860, AT1G62910, AT1G62930, AT1G63070, AT1G63080, AT1G63130, AT1G63150, AT1G63230, AT1G63330, AT1G63400, AT1G64580, AT2G41720, AT3G16710, AT4G26800, AT5G16640, AT5G41170, AT5G65560
163	163a	1.86 | -1.87	SAMT	AT1G66690, AT1G66700, AT1G66720, AT3G44860, AT3G44870
164	164a	1.15 | -2.59	NAC	AT1G56010, AT3G12977, AT3G15170, AT5G07680, AT5G53950, AT5G61430
165/166	165a<br>165b<br>166c	1.17 | 3.68<br>1.63 | 3.36<br>3.55 | -1.61	HD-ZIP III	AT1G30490, AT1G52150, AT2G34710, AT4G32880, AT5G60690
167	167d	2.72 | -3.38	ARF	-
169	169a	1.90 | 4.37	HAP2	AT1G17590, AT1G54160, AT1G72830, AT3G20910, AT5G12840
173	173a	1.64 | 5.61	TAS1,TAS2	AT1G50055, AT2G27400, AT2G39675, AT2G39681
390	390a	1.29 | 9.88	TAS3	AT3G17185
394	394a	-1.06 | 5.51	F-Box	AT1G27340
395	395a	-1.21 | 4.67	APS,AST	AT3G22890, AT4G14680, AT5G10180, AT5G43780
399	399a<br>399c	2.45 | 5.03<br>-17.71 | 8.00	E2-UBC	AT2G33770
402	402	1.10 | 2.52	ROS1-Like	AT4G34060
413	413	1.55 | 4.96	-	-
773	773	4.72 | 17.45	MET2	AT4G14140
775	775	4.00 | 22.28	GT	AT1G53290
777	777	1.23 | 3.25	CIP4.1-like	AT1G30060
779	779	2.84 | 7.39	-	AT5G53890
823	823	4.40 | 17.06	CMT3	AT1G69770
825	825	1.22 | -3.61	-	-
827	827	-1.53 | 3.39	SPX	AT1G02860
828	828	1.41 | 3.93	MYB	AT1G66370
829	829	2.85 | 10.91	-	-
850	850	-1.31 | -3.90	-	-
856	856	6.67 | 13.39	CHX	AT5G41610
861	861	1.43 | -2.31	-	-
863	863	-1.12 | -3.39	-	-
865	865	-1.08 | 2.03	-	-
3932	3932b	-1.09 | -9.87	-	-
4221	4221	2.35 | 7.06	-	-

Note. Red denotes induction by glucose after three days of light exposure; green represents repression. Mannitol and glucose fold values are in comparison to the pri-miR expression in non-treated Col-0 seedlings. Potential HXK1-dependent pri-miRs (Figure 2) are underlined. SPL genes marked with an asterisk are exclusive targets of miR156. Target gene families are based on miRBase (Kozomara and Griffiths-Jones, 2011) and TarBase (Vergoulis *et al*., 2011) databases.

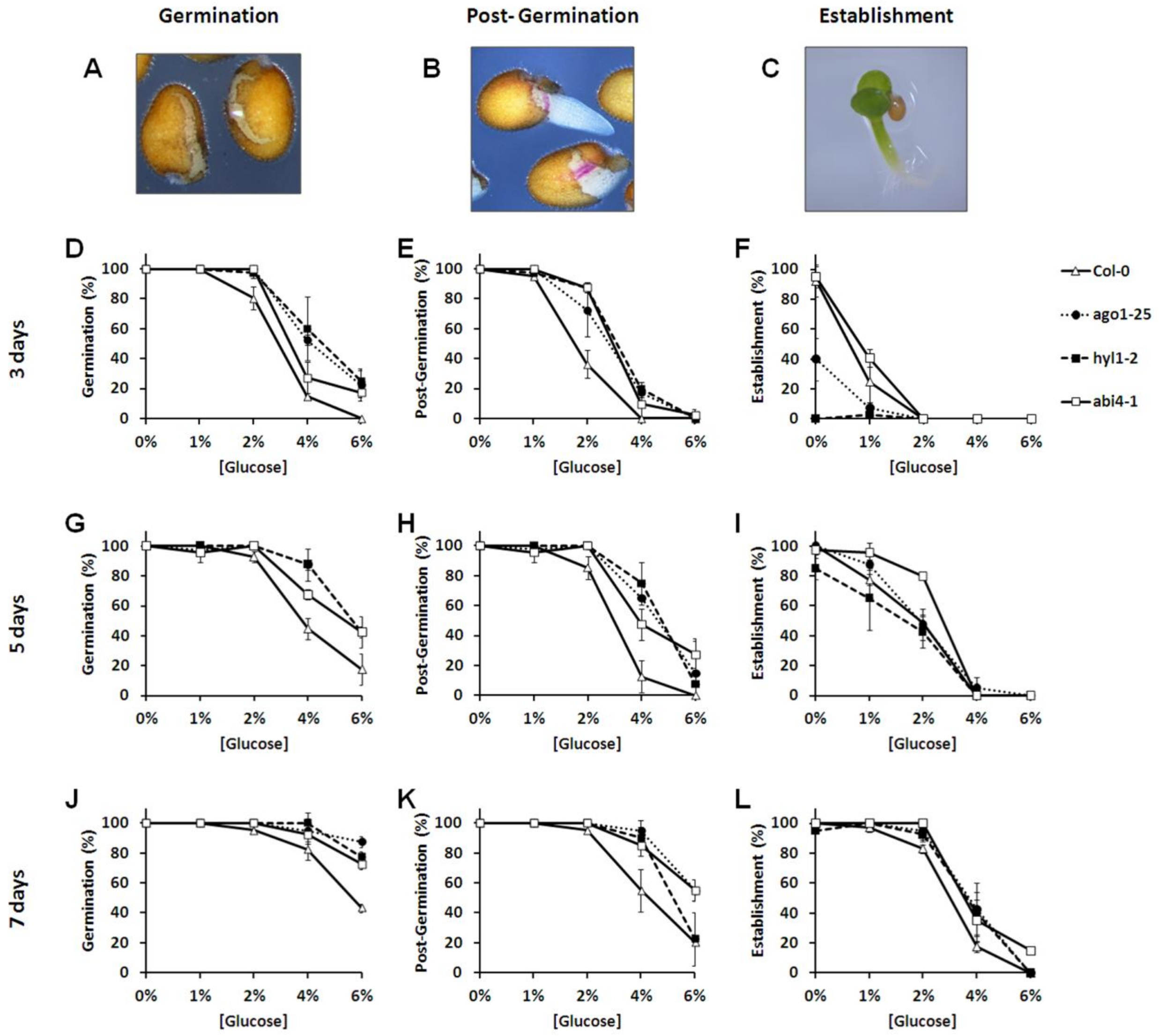

Supplementary Fig. S1. Effects of different concentrations of glucose on early seedling development of wild-type Col-0, miRNA-deficient mutants *ago1-25* and *hyl1-2*, and glucose-insensitive mutant *abi4-1*/*gin6*. (**A**) Germination (*testa* rupture), (**B**) post-germination (radicle emergence and elongation), and (**C**) establishment (cotyledons expansion and greening) were scored three days (**D-F**), five days (**G-I**) and seven days (**J-L**) after light exposure. Seeds of each genotype were sown in MS/2 plates supplied with the indicated sugar concentration, kept for two days at 4°C in the dark for stratification and transferred to continuous light (50 µmol m^{-2} s^{-1}) at 24°C. Results presented are the means ± SD of two independent experiments each of which including 20 seeds.

A

3 days

Model	Deviance	ΔDeviance	*df*	$P(\chi^2)$
Null	373.29			
Genotype	320.58	52.71	4	9.78e-11 *
Genotype + Treatment	262.40	59.18	2	2.33e_13 *
Genotype + Treatment + (Genotype x Treatment)	228.48	33.92	8	4.19e-05 *

5 days

Model	Deviance	ΔDeviance	*df*	$P(\chi^2)$
Null	157.81			
Genotype	134.12	23.69	4	9.21e-05 *
Genotype + Treatment	125.66	8.46	2	0.014 *
Genotype + Treatment + (Genotype x Treatment)	111.86	13.80	8	0.087

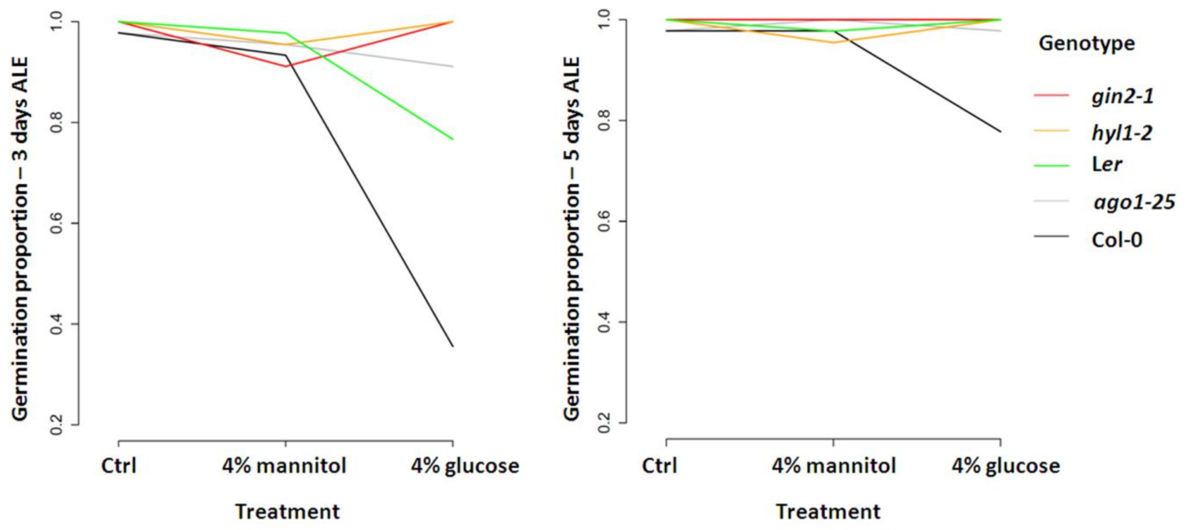

Model	Deviance	ΔDeviance	*df*	$P(\chi^2)$
Null	509.95			
Genotype	454.07	55.88	4	2.13e-11 *
Genotype + Treatment	337.33	116.74	2	<2.2e-16 *
Genotype + Treatment + (Genotype x Treatment)	299.21	38.13	8	7.14e-06 *

Model	Deviance	ΔDeviance	*df*	$P(\chi^2)$
Null	279.09			
Genotype	219.88	59.21	4	4.24e-12 *
Genotype + Treatment	171.26	48.61	2	2.78e-11 *
Genotype + Treatment + (Genotype x Treatment)	153.16	18.11	8	0.0204 *

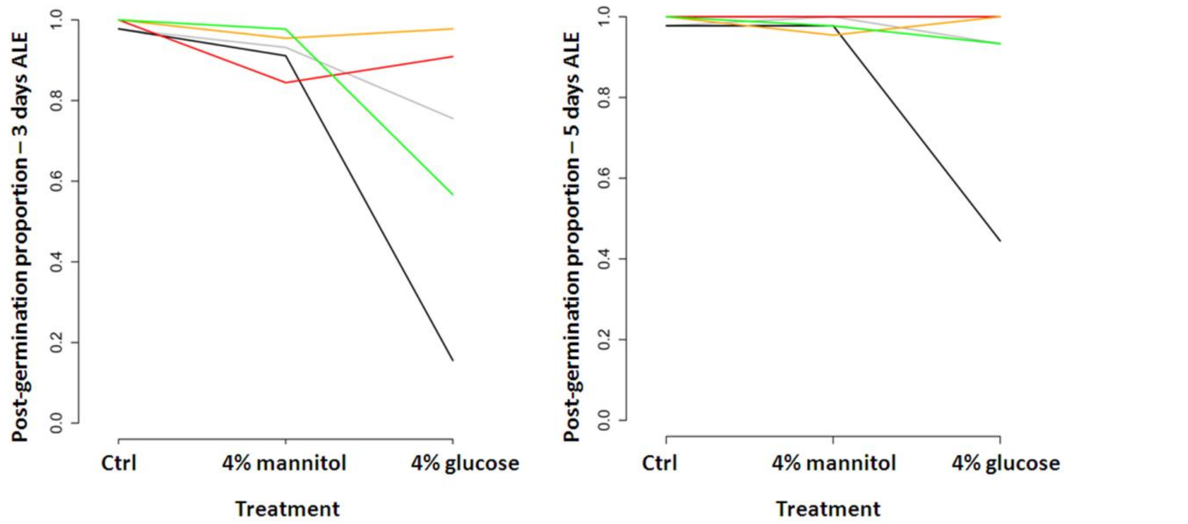

Model	Deviance	ΔDeviance	*df*	$P(\chi^2)$
Null	817.19			
Genotype	792.23	24.95	4	5.14e-05 *
Genotype + Treatment	622.80	169.44	2	<2.2e-16 *
Genotype + Treatment + (Genotype x Treatment)	556.76	66.04	8	3.01e-11 *

Model	Deviance	ΔDeviance	*df*	$P(\chi^2)$
Null	474.76			
Genotype	400.21	74.55	4	2.49e-15 *
Genotype + Treatment	266.73	133.48	2	<2.2e-16 *
Genotype + Treatment + (Genotype x Treatment)	242.91	23.82	8	0.0025 *

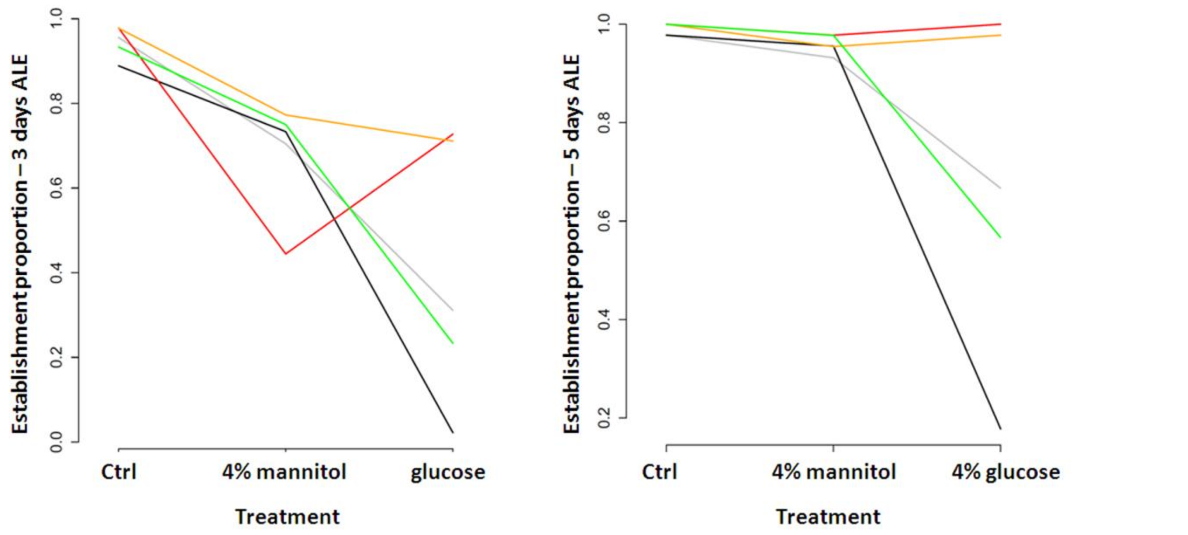

Supplementary Fig. S2. Analyses of deviance and interaction plots used to compare the germination and development efficiencies of each genotype grown in the following conditions: **(A)** Three or five days after light exposure in control media or media supplied with 4% glucose or 4% mannitol (Fig. 1 samples); **(B)** Three or five days after light exposure in control media or media supplied with 4% glucose or 4% mannitol (Supplementary Fig. S3 samples); **(C)** Five days after light exposure in control media or media supplied with 0.5-5 µM of ABA (Supplementary Fig. S4 samples). The null model considered no effect of treatment or genotype in germination or development efficiencies, nor interaction between these variables. Significant *p* values ($P < 0.05$) are marked with an asterisk.

B

3 days

Model	Deviance	Δ Deviance	*df*	*P* (χ^2)
Null	348.80			
Genotype	326.35	22.45	4	1.63e-04 *
Genotype + Treatment	276.00	50.35	2	1.17e-11 *
Genotype + Treatment + (Genotype x Treatment)	249.32	26.68	8	8.02e-04 *

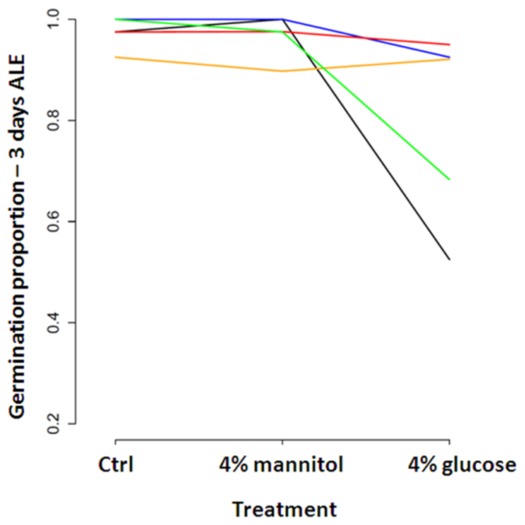

5 days

Model	Deviance	Δ Deviance	*df*	*P* (χ^2)
Null	140.24			
Genotype	123.08	17.16	4	1.8e-03 *
Genotype + Treatment	121.81	1.27	2	0.529
Genotype + Treatment + (Genotype x Treatment)	105.38	16.43	8	0.037 *

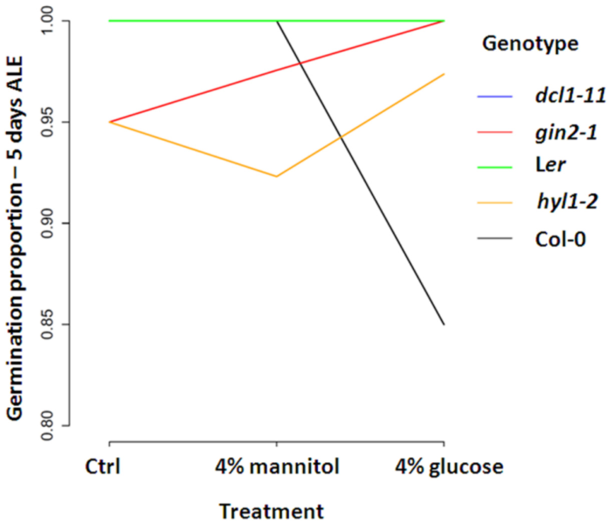

Model	Deviance	Δ Deviance	*df*	*P* (χ^2)
Null	503.45			
Genotype	470.91	32.54	4	1.48e-06 *
Genotype + Treatment	323.32	147.59	2	< 2.2e-16 *
Genotype + Treatment + (Genotype x Treatment)	276.77	46.54	8	1.87e-07 *

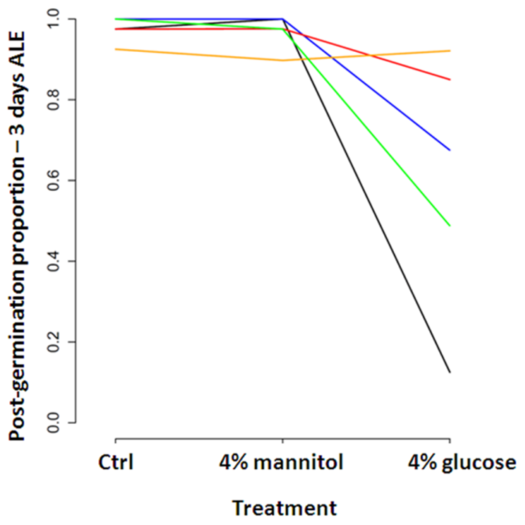

Model	Deviance	Δ Deviance	*df*	*P* (χ^2)
Null	255.47			
Genotype	228.75	26.72	4	2.27e-05 *
Genotype + Treatment	200.38	28.37	2	6.92e-07 *
Genotype + Treatment + (Genotype x Treatment)	164.26	36.11	8	1.67e-05 *

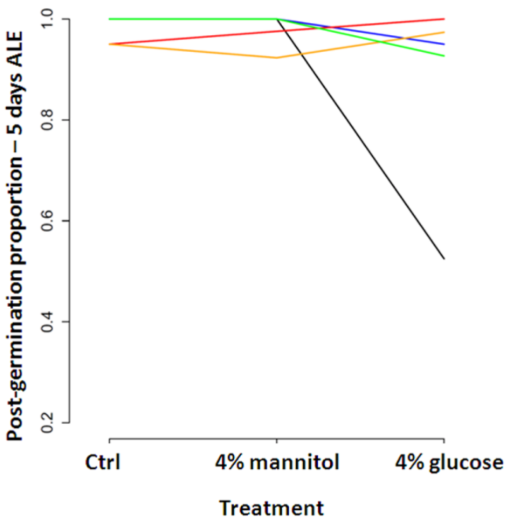

Model	Deviance	Δ Deviance	*df*	*P* (χ^2)
Null	708.99			
Genotype	684.60	24.39	4	6.66e-05 *
Genotype + Treatment	454.09	230.51	2	< 2.2e-16 *
Genotype + Treatment + (Genotype x Treatment)	389.44	64.64	8	5.68e-11 *

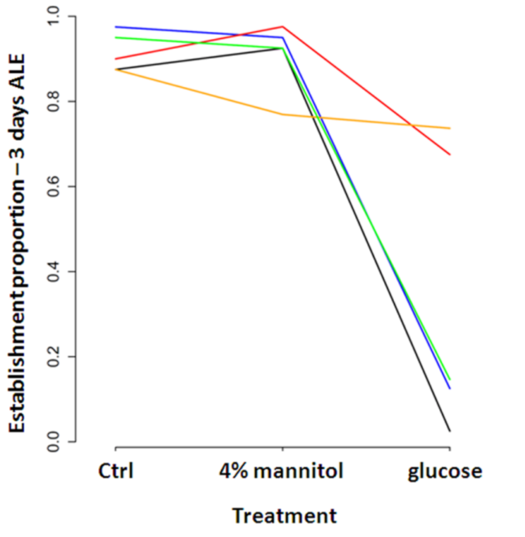

Model	Deviance	Δ Deviance	*df*	*P* (χ^2)
Null	432.03			
Genotype	393.13	38.90	4	7.3e-08 *
Genotype + Treatment	282.52	110.61	2	< 2.2e-16 *
Genotype + Treatment + (Genotype x Treatment)	239.54	42.97	8	8.89e-07 *

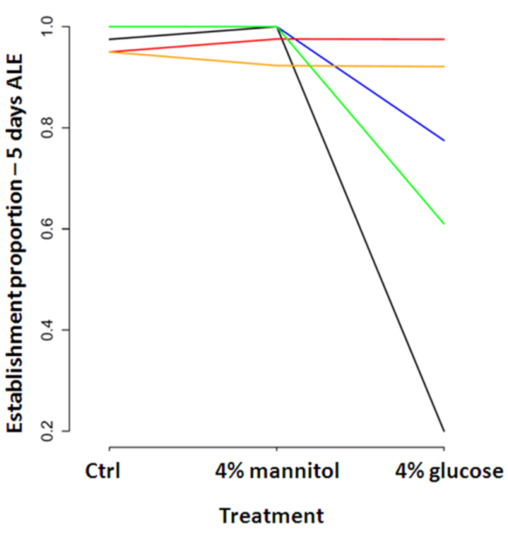

Supplementary Fig. S2. Continued.

C

5 days

Model	Deviance	Δ Deviance	*df*	*P* (χ^2)
Null	348.80			
Genotype	326.35	22.45	4	1.63e-04 *
Genotype + Treatment	276.00	50.35	2	1.17e-11 *
Genotype + Treatment + (Genotype x Treatment)	249.32	26.68	8	8.02e-04 *

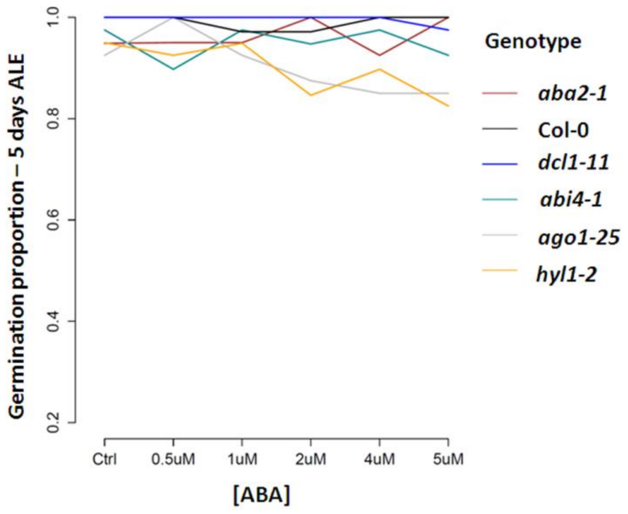

Model	Deviance	Δ Deviance	*df*	*P* (χ^2)
Null	503.45			
Genotype	470.91	32.54	4	1.48e-06 *
Genotype + Treatment	323.32	147.59	2	< 2.2e-16 *
Genotype + Treatment + (Genotype x Treatment)	276.77	46.54	8	1.87e-07 *

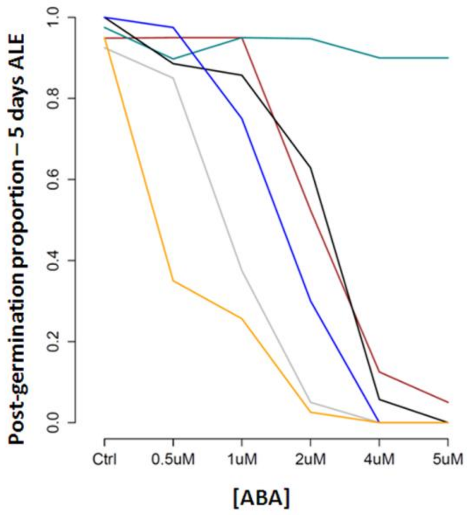

Model	Deviance	Δ Deviance	*df*	*P* (χ^2)
Null	708.99			
Genotype	684.60	24.39	4	6.66e-05 *
Genotype + Treatment	454.09	230.51	2	< 2.2e-16 *
Genotype + Treatment + (Genotype x Treatment)	389.44	64.64	8	5.68e-11 *

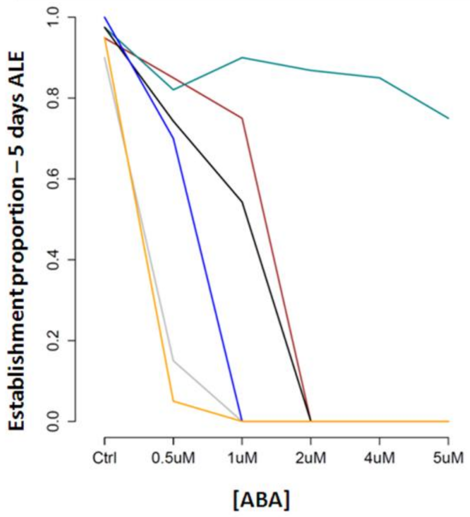

Supplementary Fig. S2. Continued.

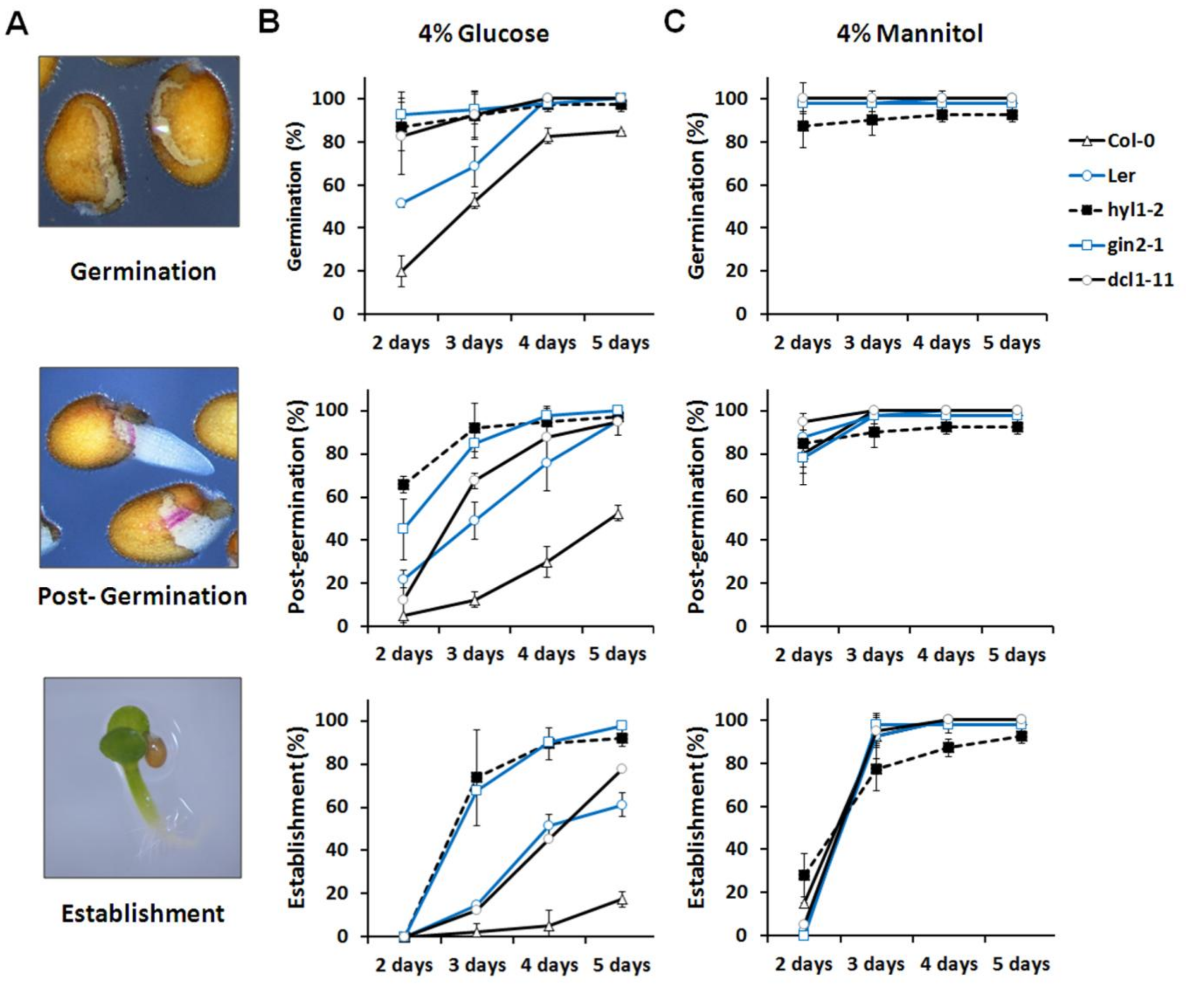

Supplementary Fig. S3. Glucose-induced delay of early seedling development is dependent upon miRNA machinery activity **(A)** Developmental phases that were monitored; Germination (*testa* rupture), post-germination (radicle emergence and elongation) and establishment (cotyledons expansion and greening); **(B)** Effects of 4% glucose on germination and development of miRNA-deficient mutants *dcl1-11* and *hyl1-2* was less severe than for wild-type Col-0 and L*er*, and was similar to the glucose insensitive *gin2-1*. **(C)** Osmotic control 4% mannitol could not reproduce the delay observed for glucose. In media not supplied with sugar, all seeds reached post-germination stage after two days, and establishment within three days after light exposure (Supplementary Fig. S2B). In glucose-supplied media, growth arrest was not observed before seedling establishment stage was reached. Seeds of each genotype were sown in MS/2 plates supplied or not with the indicated sugar, kept for two days at 4°C in the dark for stratification and transferred to continuous light (50 µmol m^{-2} s^{-1}) at 24°C. Germination, post-germination, and establishment were scored from two to five days after light exposure. Results presented are the means ± SD of three independent experiments each of which including 20 seeds.

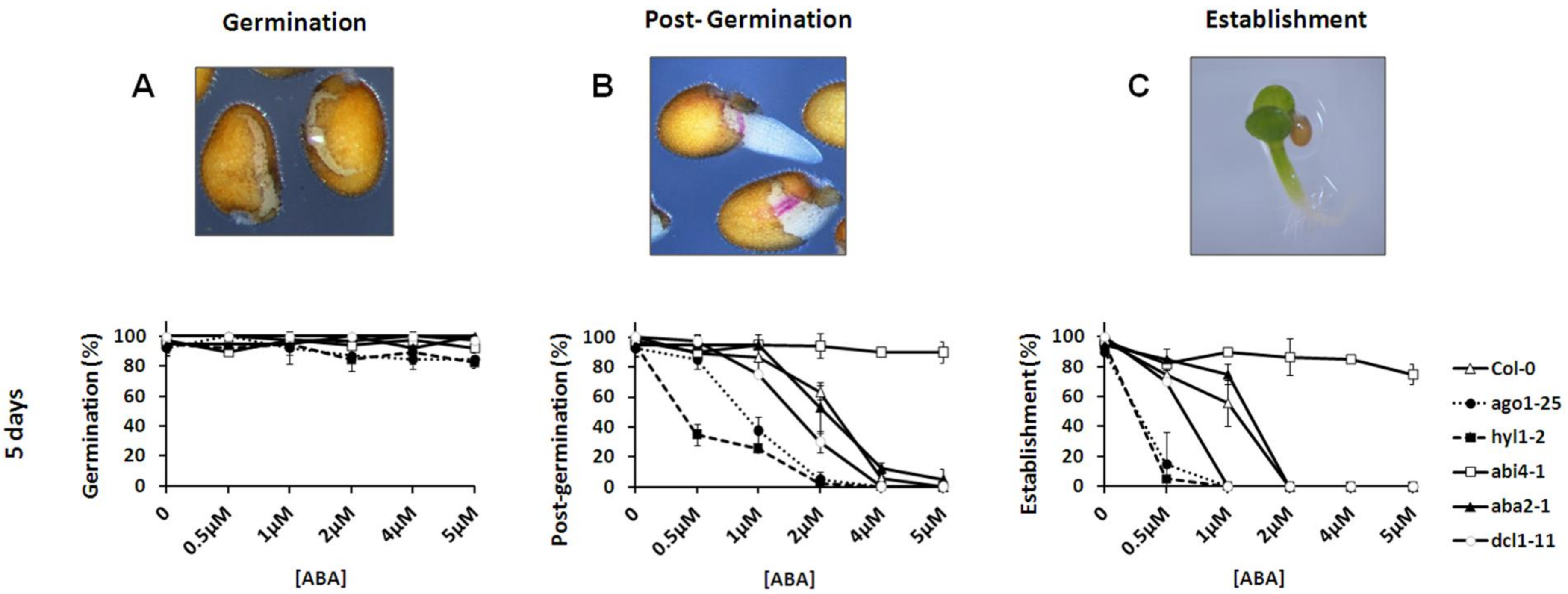

Supplementary Fig. S4. Effects of different ABA concentrations on early seedling development of wild-type Col-0, miRNA-deficient mutants *ago1-25*, *dcl1-11* and *hyl1-2*, ABA-insensitive mutant *abi4-1*, and ABA-biosynthesis mutant *aba2-1*. **(A)** Germination (*testa* rupture), **(B)** post-germination (radicle emergence and elongation) and **(C)** establishment (cotyledons expansion and greening) were scored five days after light exposure. Except for the ABA-insensitive mutant *abi4-1*, all genotypes had the development arrested before establishment within five days (Supplementary Fig. S2C). Seeds of each genotype were sown in MS/2 plates supplied with the indicated ABA concentration, kept for two days at 4°C in the dark for stratification and transferred to continuous light (50 µmol m^{-2} s^{-1}) at 24°C. Results presented are the means ± SD of two independent experiments each of which including 20 seeds.

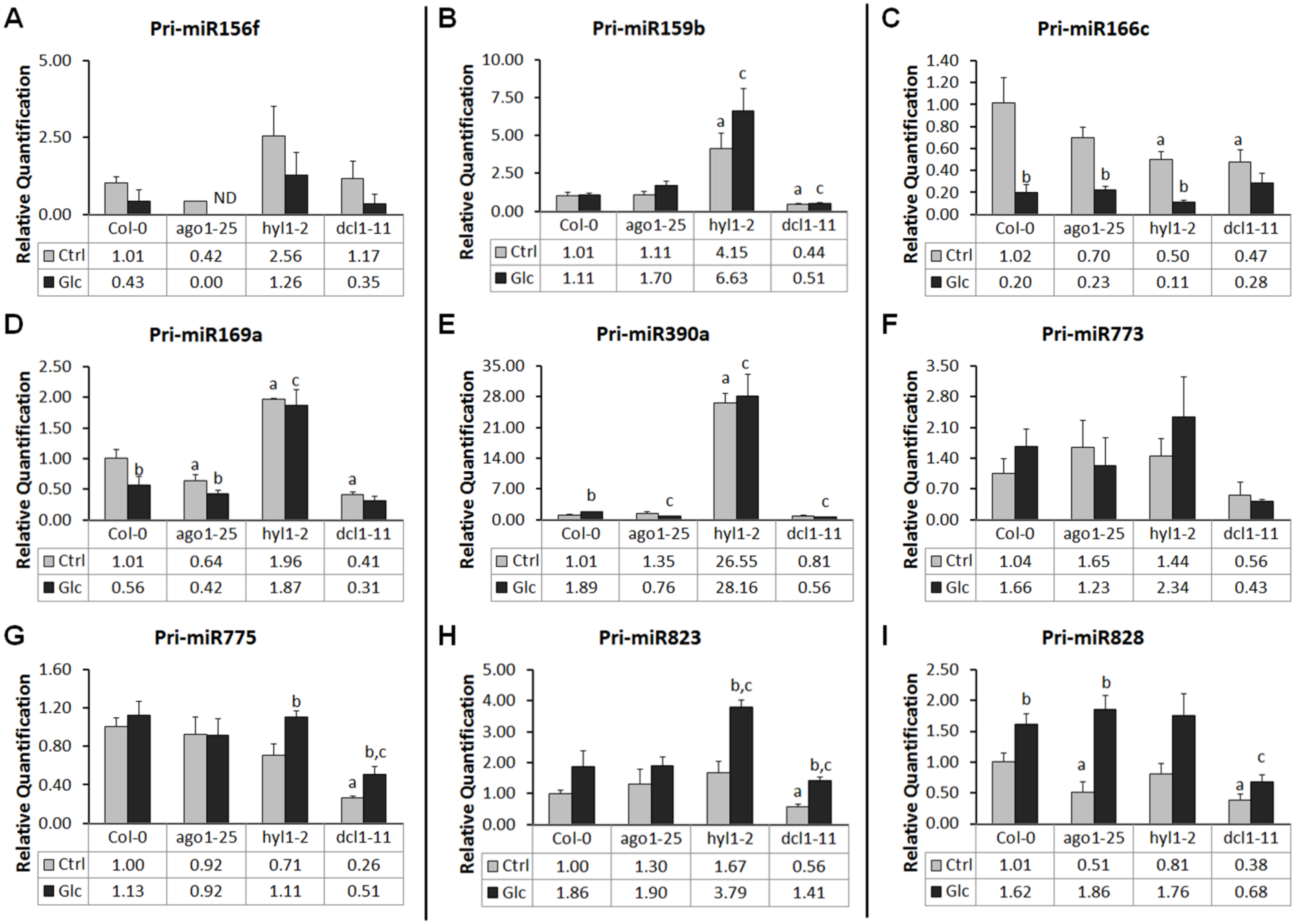

Supplementary Fig. S5. Accumulation of **(A)** pri-miR156f, **(B)** pri-miR159b, **(C)** pri-miR166c, **(D)** pri-miR169a, **(E)** pri-miR390a, **(F)** pri-miR773, **(G)** pri-miR775, **(H)** pri-miR823, and **(I)** pri-miR828 in Col-0, *ago1-25*, *dcl1-11* and *hyl1-2* seedlings grown in control media for three days of light exposure and treated for 4h with 4% glucose. All expression values are in comparison to untreated Col-0. The values are means of three biological replicates ± SD. Below each graph is given the relative transcript abundance. Changes in transcript accumulation were considered significant for differences with fold change ≥ |1.5| and according to Student's *t* test ($p < 0.05$) for the following comparisons: **a.** untreated *ago1-25*, *dcl1-11* or *hyl1-2 vs* untreated Col-0; **b.** glucose *vs* untreated samples (same genotype); **c.** glucose-treated mutant *vs* glucose-treated.

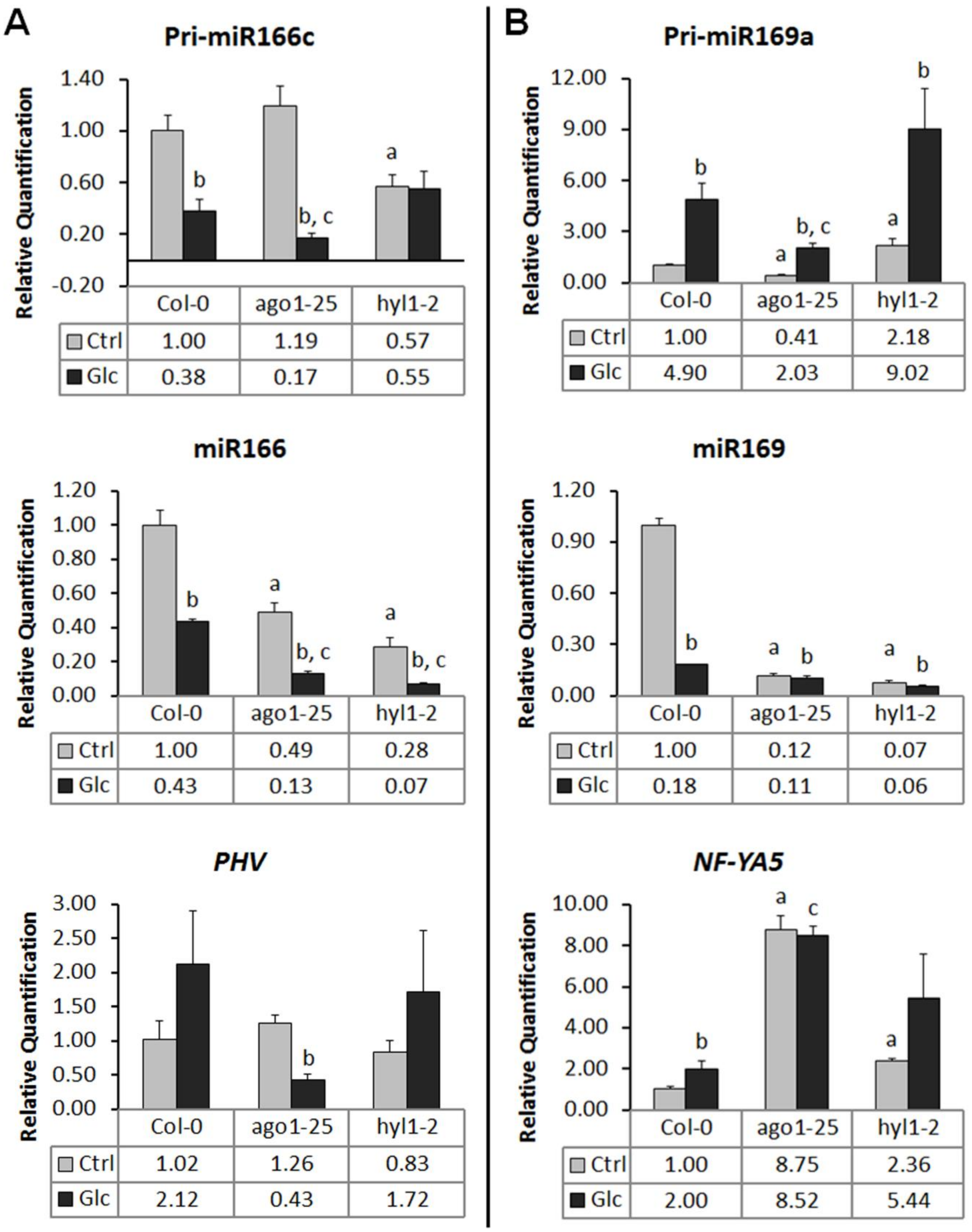

Supplementary Fig. S6. Accumulation of **(A)** pri-miR166c, **(B)** pri-miR169a, **(C)** pri-miR390a, **(D)** pri-miR773, **(E)** pri-miR775, **(F)** pri-miR823, **(G)** pri-miR828 and their corresponding mature miRNA family and respective targets in Col-0, *ago1-25* and *hyl1-2* seedlings grown in 4% glucose or control media after three days of light exposure. All expression values are in comparison to untreated Col-0. The values are means of three biological replicates ± SD. Below each graph is given the relative transcript abundance. Changes in transcript accumulation were considered significant for differences with fold change ≥ |1.5| and according to Student's *t* test ($p < 0.05$) for the following comparisons: **a.** untreated *ago1-25* or *hyl1-2* *vs* untreated Col-0; **b.** glucose *vs* untreated samples (same genotype); **c.** glucose-treated mutant *vs* glucose-treated Col-0. miR828 did not yield a specific amplification.

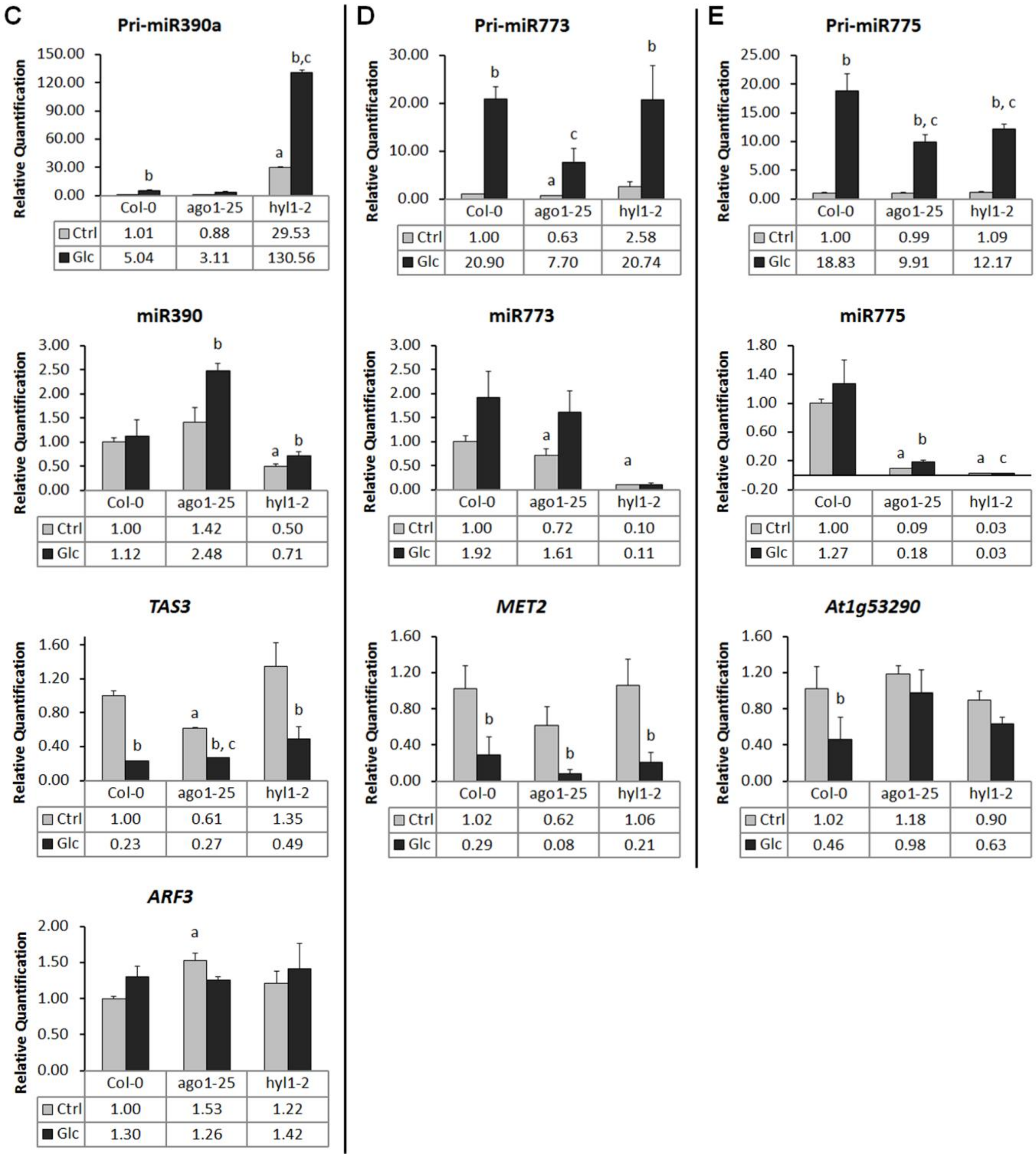

Supplementary Fig. S6. Continued.

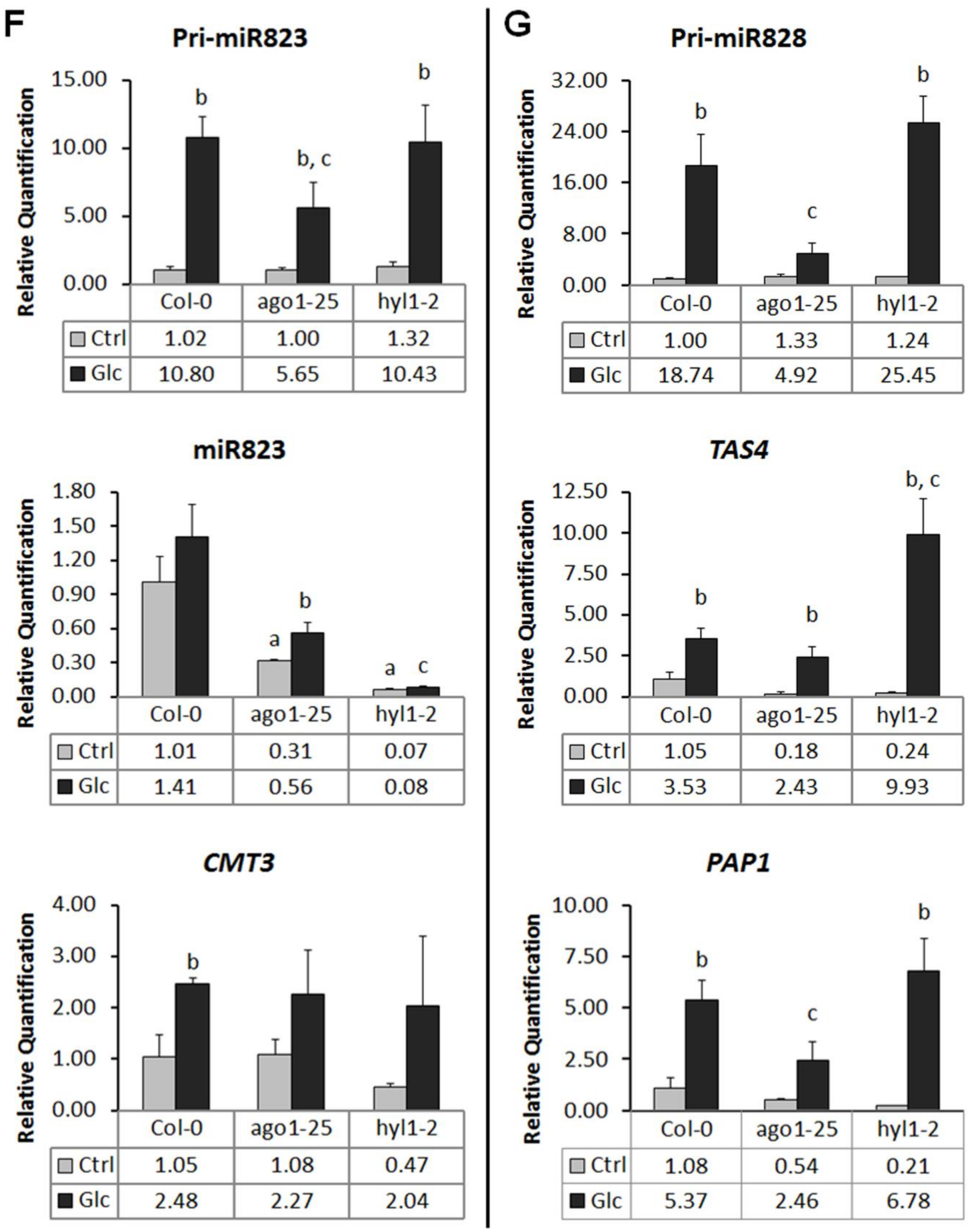

Supplementary Fig. S6. Continued.

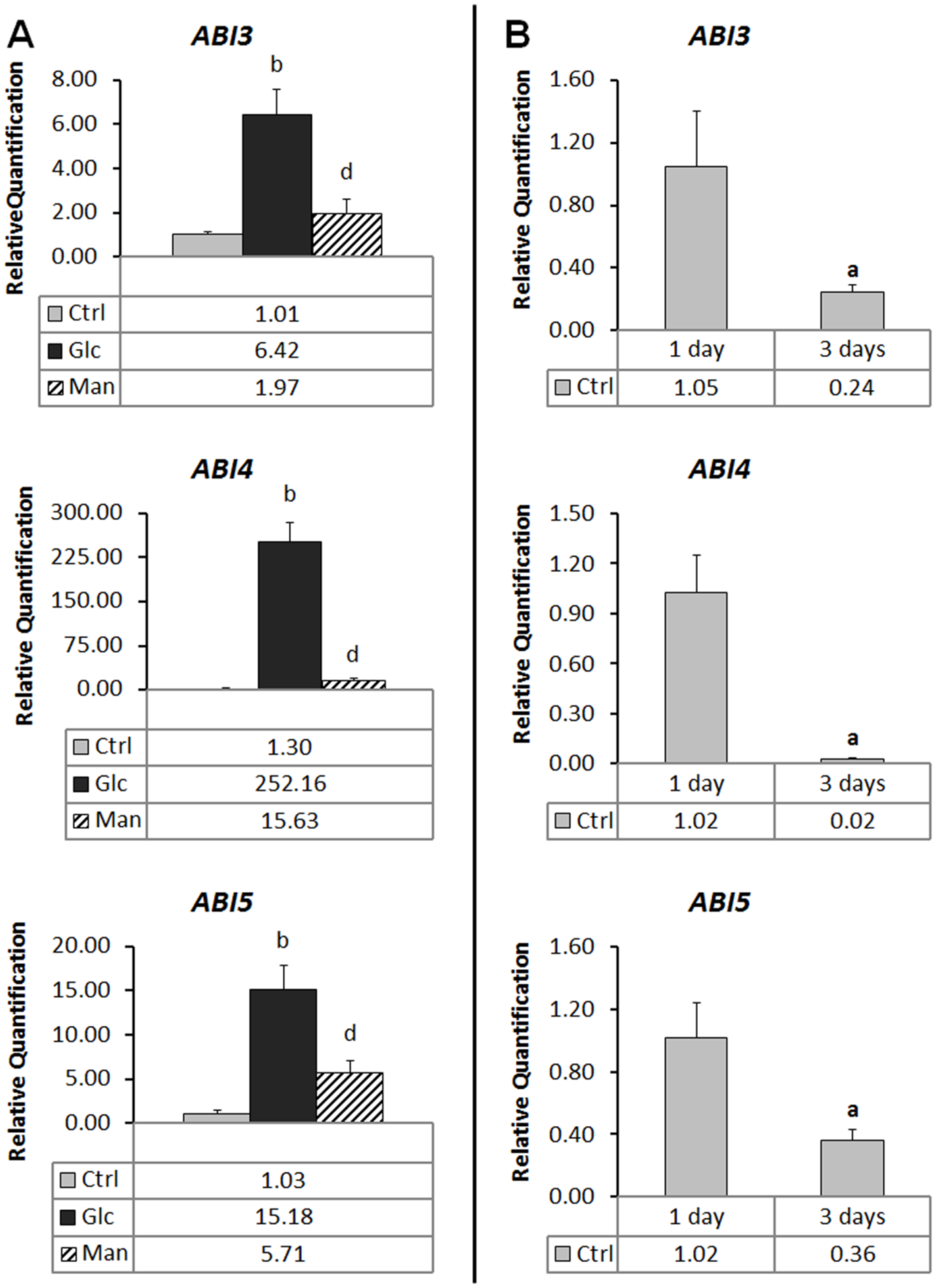

Supplementary Fig. S7. Validation of glucose-promoted changes on *ABI3*, *ABI4* and *ABI5* accumulation. **(A)** Relative expression of *ABI3*, *ABI4* and *ABI5* in Col-0 seedlings grown in 4% glucose or 4% mannitol in comparison to untreated samples three days after light exposure (ALE). **(B)** Relative expression of *ABI3*, *ABI4* and *ABI5* in untreated Col-0 seedlings with one day ALE in comparison to three days ALE samples. The values are means of three biological replicates ± SD. Below each graph is given the relative transcript abundance. Changes in transcript accumulation were considered significant for differences with fold change ≥ |1.5| and according to Student's *t* test ($p < 0.05$) for the following comparisons: **a.** untreated one day ALE *vs* three days ALE samples; **b.** glucose *vs* untreated samples; **d.** mannitol- *vs* glucose-treated samples.
